# Gaps in the evidence on improving social care outcomes: findings from a meta‐review of systematic reviews

**DOI:** 10.1111/hsc.12300

**Published:** 2015-10-26

**Authors:** Kelly Dickson, Katy Sutcliffe, Rebecca Rees, James Thomas

**Affiliations:** ^1^ Evidence for Policy and Practice Information and Co‐ordinating Centre (EPPI‐Centre)Social Science Research UnitUCL Institute of EducationLondonUK

**Keywords:** evaluating complex interventions, health and social care, policy research, quality of life, trials

## Abstract

Adult social care continues to be a central policy concern in the UK. The Adult Social Care Outcomes Framework (ASCOF) is a range of measures nationally available to drive forward improvement on outcomes and quality in local councils. While there is an emphasis on improving transparency, quality and outcomes, drawing on research evidence to achieve these aims is often difficult because the evidence is not easily identifiable, is disparate or of variable quality. We conducted a meta‐review to analyse and summarise systematic review‐level evidence on the impact of interventions on the four outcomes set out in the ASCOF: quality of life, delaying and reducing the need for services, satisfaction with services and safeguarding of vulnerable adults. This paper focuses on the availability of review‐level evidence and the presence of significant gaps in this evidence base. A range of health and social care databases were searched, including MEDLINE, ASSIA and The Cochrane Library in January and February 2012. All systematic reviews evaluating the efficacy of social care interventions for improving ASCOF outcomes for older people, people with long‐term conditions, mental health problems or physical and/or learning disabilities were eligible. Two reviewers independently screened systematic reviews for quality and relevance and extracted data; 43 systematic reviews were included, the majority of which examined the impact of interventions on quality of life (*n* = 34) and delaying and reducing the need for support (*n* = 25). Limited systematic review‐level evidence was found regarding satisfaction with services and safeguarding. There were also significant gaps in relation to key social care interventions and population groups. Research priorities include addressing these gaps and the collation of data on interventions, outcomes and populations more closely related to social care. Overall, a more relevant, comprehensive and robust evidence base is required to support improvement of outcomes for recipients of adult social care.

**What is known about this topic**

- There is a policy drive to improve adult social care outcomes in the UK.
- Systematically identifying an evidence base on the effectiveness of social care interventions is challenging because of a lack of agreed definitions in this diverse area.

**What this paper adds**

- Despite drawing on review‐level data to support the inclusion of a breadth of interventions, population groups, outcome‐based evidence on many social care initiatives relevant to the UK were not found, and therefore need to be reviewed.
- However, the large body of evidence that was found on the effectiveness of physical activity for people with long‐term conditions and non‐frail older people may be relatively cheap and easy to implement, and therefore worth considering.

## Introduction

Developing an evidence base and the role of evidence‐based policy and practice in social care has continued to expand in the past decade. This can be witnessed in the UK by the establishment of organising bodies such as the National Institute of Clinical Excellence (NICE) Collaborating Centre for Social Care and The School for Social Care Research, funded by the National Institute for Health Research. A key policy driver was the move towards modernising social services (Department of Health 1998, DH 2001a) and a commitment to enhancing the contribution of research in health and social care (DH 2001b). Taking account of different types of evidence suitable to answering a range of policy and practice relevant questions has always been central to the aims of evidence‐based social care (Pawson *et al*. 2003, Marsh & Fisher 2005, Fisher 2014). Further focus is now being placed on the effectiveness of social care interventions and the need to identify and measure outcomes that can sensitively capture the differences between social care populations, interventions and intended impacts in the short and long term (Malley & Netten 2009).

In November 2010, the UK government consultation document ‘Transparency in outcomes: A framework for adult social care’ (DH 2010a) was published alongside its major policy statement ‘A vision for adult social care: capable communities and active citizens’ (DH 2010b) setting out a new strategy for achieving transparency, quality and outcomes in adult social care. A key element of the government's approach to accountability in the social care system was the development of the Adult Social Care Outcomes Framework (ASCOF); with an updated version of the ASCOF published in 2012 (DH 2012). The ASCOF is a group of measures, nationally available to councils in England, to support a drive towards improvement on outcomes and quality at the local level. In order to build a view of the evidence to influence the structure and development of the ASCOF, there was a need to review existing evidence on the effectiveness of social care interventions and to bring this together in a transparent way to support policy decision‐making.

### Aims

This paper reports on a piece of research, commissioned by the Department of Health and conducted by the EPPI‐Centre, that synthesised review‐level evidence on adult social care interventions, and the extent to which the outcomes set out in the ASCOF can be improved and which interventions are most effective for doing this. The research comprised a meta‐review of systematic reviews to answer the following question:

*Which social care interventions can effectively improve outcomes for services users in the four outcome domains set out in the ASCOF: quality of life (QoL), prevention, satisfaction and safeguarding?*

The aim of this paper was to provide a summary of the evidence found and highlight the significant gaps identified. The comprehensive account of the approach and findings with further contextual detail are contained in the full technical report (Sutcliffe *et al*. 2012).

### Design and scope

We conducted a meta‐review of systematic reviews; (i.e. where findings of systematic reviews are examined rather than individual primary studies, sometimes referred to as ‘reviews of reviews’, ‘overviews of reviews’ or ‘umbrella reviews’) adhering (PRISMA) guidance (see S1). Meta‐reviews are considered an appropriate review tool to inform policy and practice when a topic area is particularly broad or when it is already populated with many systematic reviews (Caird *et al*. 2015). Conducting a meta‐review of what is known about the effects of social care interventions has enabled the possibility of producing a synthesis of available evidence covering a range of social care interventions, populations and outcomes in a way that a systematic review focusing on a single social care intervention and/or population could not. By making the available evidence accessible, our goal was to assist policy makers and other stakeholders make decisions and improvements in social care and to reduce potential uncertainty when deciphering conflicting findings across related reviews that often vary in size and quality. This meta‐review also helped to identify evidence gaps, such as areas requiring updated systematic reviews and the need for further evaluations (Becker & Oxman 2008).

Systematic and meta‐reviews are able to address a range of important policy and practice concerns, including issues of implementation and receipt of social care interventions, making use of different types of research appropriate to answering those questions (e.g. qualitative synthesis of stakeholder perspectives of personal budgets Fleming *et al*. 2015, older people's experiences of receiving social care, São José *et al*. 2015). For this meta‐review, we were asked by the Department of Health to investigate the ability of social care interventions to improve outcomes as defined in the adult social care framework, and as such we drew together evidence from reviews of experimental studies as they are widely acknowledged as the most appropriate for determining effectiveness (Gough *et al*. 2012).

### Concepts and definitions

Definitions of adult social care interventions, population groups and outcomes guiding this meta‐review are outlined in Table 1. Transparently defining the phenomena of interest enabled us to act systematically when screening reviews for eligibility and to ensure the scope of this meta‐review is communicated clearly. However, a major challenge for this meta‐review is its attempts to systematically address a diverse and complex area lacking commonly agreed definitions (Law Commission 2011).

**Table 1:** Adult social care framework (adapted from DH 2010a)

Key concepts	Definitions
Adult Social Care Interventions	Interventions needed to be led by or completely provided by someone other than a health professional, and have the aim of supporting activities of daily living, or preventing an increased need for services, rather than treating a condition
Population groups:	
Older people	People aged 65 years and over
Adults with mental health problems	People aged 18 years or over with a diagnosed mental health problem, disorder or disability – including substance misuse and other addictions
Adults with physical disabilities	People aged 18 years or over with a physical impairment which has a substantial and long‐term effect on their ability to carry our day‐to‐day activities
Adults with learning disabilities	People aged 18 years or over with a learning disability/intellectual impairment which has a substantial effect on their ability to carry our day‐to‐day activities
Adult Social Care Outcomes:	
Quality of life‘Enhancing quality of life for people with care and support needs’ (p. 27)	Within quality of life we aimed to capture measures of ☐ Overall quality of life (QoL) As well as some more specific quality of life measures ☐ Being able to take part in the activities of daily living (ADL) ☐ Being able to participate in social activities such as employment (social participation) ☐ Feeling safe or having a sense of control or dignity (dignity/control)
Prevention:	
‘Delaying and reducing the need for care and support’ (p. 27)	Within prevention we capture both ☐ Direct measures of increased need for the use of health or social care services, such as time spent in hospital (service use) ☐ Measures of illness or events, such as falls, which could lead to an increased need for services (illness/events)
Satisfaction with services: ‘ensuring that people have a positive experience of care and support’ (p. 27)	Satisfaction with services included: ☐ Service users’ general satisfaction with care and support services ☐ Experiences of information and advice services ☐ Perceptions of whether services respect dignity and are sensitive to individual circumstances and preferences
Safeguarding: ‘safeguarding adults whose circumstances make them vulnerable and protecting from avoidable harm’ (p. 27)	Safeguarding relates to protection of adults who ☐ Have health or social care needs ☐ Are at risk of significant harm ☐ Are unable to safeguard themselves as a result of their health or social care needs

The diversity and complexity of social care interventions is inherent in their design. They range from practical help and equipment to emotional support, such as befriending schemes or occupational therapy. Interventions typically involve a number of components which may act both independently and interdependently (MRC 2000) and may be further influenced by being delivered by different individuals, at different intensities and in different settings. Moreover, recent policy developments in the UK have increased the complexity of social care services through the personalisation agenda, which means that social care is now seen as including not only standard ‘off the shelf’ services but also care packages chosen or created by care recipients. This new vision of social care also includes initiatives aiming to reduce the need for social care through more effective provision of transport, leisure and other so‐called ‘universal services’. The increased focus on integrating health and social care that makes it difficult, or even sometimes of questionable value, to identify the contribution of social care practices as distinct from health services (Law Commission 2011). Understanding of the evidence on social care interventions is further complicated by differences in the organisation, delivery and practice of social care between countries. In terms of definitions, there is a lack of agreement, even within the UK, as to what constitutes social care or social care populations. Similarly, the ASCOF framework makes reference to various kinds of potential social care outcome, but aims to illustrate, rather than provide watertight definitions.

## Methods

### Search

The search took place between January and February 2012, when the review was commissioned, using the following bibliographic databases: The Cochrane Library, Database of Abstracts of Reviews of Effects, Health Technology Assessment, National Health Service Economic Evaluation Database, Pubmed, Embase, PsychInfo, ASSIA, Social Science Citation Index, International Bibliography of the Social Sciences, Sociological Abstracts & Social Services Abstracts, and Social Care Online. These were supplemented with hand searches and reference list checking of all systematic reviews (see S1).

### Screening

Systematic reviews were included if they synthesised or summarised evidence about the impact of social care interventions for adults on one or more of the four ASCOF outcomes. Reviews from non‐OECD countries were excluded on the basis that their contexts were not considered directly comparable to social care systems in developed countries. Reviews needed to be published in English from 2007 onwards to ensure we focused on the most up‐to‐date reviews. This was also based on the assumption that earlier reviews are likely to have been published after 2006 in an updated form. References were screened on title and abstract and then on full report. At each stage, reviewers independently screened studies in pairs only moving to screening by a single reviewer once a 90% agreement rate had been achieved.

### Quality appraisal

The quality assessment tool used two items from Elliot *et al*. (2001); use of a comprehensive search strategy of at least two bibliographic databases and use of explicit inclusion criteria in the review's methods section. Only reviews meeting this minimum quality threshold were included (Caird *et al*. 2015). To further enhance the validity of our synthesis, evidence from the included reviews was only used when based on evidence from randomised or non‐randomised trials. Judgements about the quality of these trials, as reported by the authors, were also captured and taken into account during the synthesis (see S1).

### Data extraction

A review‐specific tool was devised to extract information on the aims and findings of included reviews. Reviewers extracted review authors findings, as reported, in the form of numerical or narrative summary statements. Reviewers identified the numbers of trials used to create summary statements, and reviewed the authors’ statements about the quality of these trials, their claims about impact and recorded whether they agreed with, or had concerns about the review authors’ conclusions (See S1). Summary statements were only captured for ASCOF outcomes, based on RCTs and nRCTs. This meant that in some cases, reviewers extracted only part of the evidence from a systematic review, or extracted evidence related to just one relevant trial. Data extraction was conducted independently by two reviewers, who met to compare their work. Disagreements were resolved through discussion and arbitration of a third party where required.

### Synthesis

A narrative synthesis of the findings was conducted by examining the direction of findings and the extent and quality of the included studies as judged by the reviewers. Although we considered statistical difference, the direction of effect was our primary consideration when interpreting and synthesising results as studies not reaching statistical significance may have been insufficiently powered to detect a small, but operationally significant effect. The findings were categorised as follows: (i) evidence of positive impact: when the direction of positive effect was statistically significant; (ii) no evidence of difference: when it was not possible to detect any statistically significant differences in the direction of effect between those receiving social care interventions and those in control or comparison groups for particular outcomes (To reiterate, this lack of difference may be because the study was not large enough to detect any differences that there might have been between groups or that the intervention actually had no effect. The statement does not indicate an absence of evidence nor does it indicate equivalence between comparison groups.); (iii) evidence of harm: when the direction of effect was negative, statistically or non‐statistically; (iv) inconsistent evidence: when there were conflicting findings among studies, i.e. some find evidence of positive impact, while others found no evidence of difference; and (v) insufficient evidence: when findings were based on a single study or on two studies of poor quality. The final stage of synthesis involved bringing together the findings of multiple reviews for each intervention and outcome combination; overlapping primary studies across reviews were identified to avoid bias through ‘double counting’ of the evidence.

## Results

### Search results

**Figure 1:**
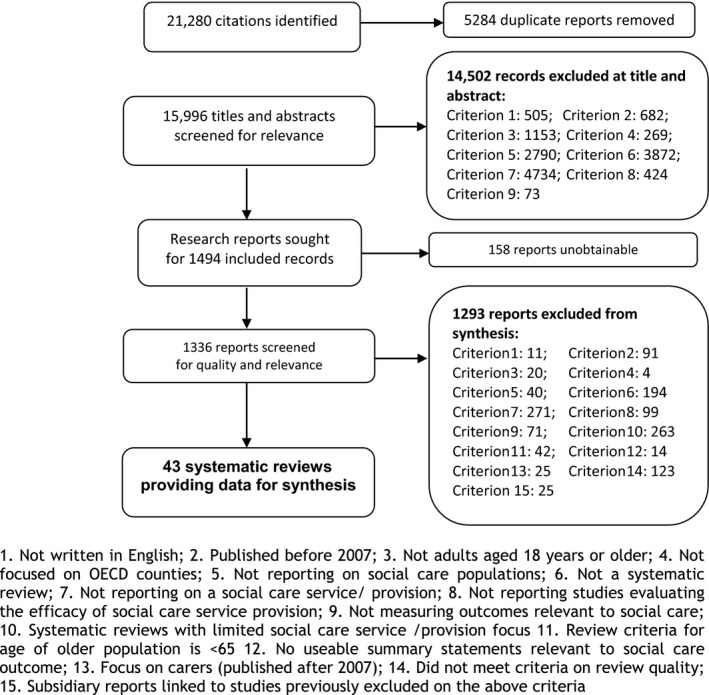
Results of searching and screening.

Figure 1 shows the results of searching and screening. After removing 5284 duplicates, 15,996 titles and abstracts were screened for relevance and quality. The majority of papers were excluded at this stage (*n* = 14,502). Full reports were retrieved (*n* = 1366) with a further 1293 papers excluded. A total of 43 systematic reviews were included in the meta‐review.

### Description of included systematic reviews

#### Outcomes, interventions and populations examined in included reviews

Although the included systematic reviews covered a range of outcomes, interventions and population groups, many were found to cluster in particular areas (Table 2). The largest group of reviews, almost 80% (*n* = 34), examined the impact of interventions on quality of life, followed by reviews measuring outcomes relevant to preventing the need for support (*n* = 25). Far fewer reviews examined users’ satisfaction with the services they received (*n* = 4) and just one review examined safeguarding outcomes.

**Table 2:** Breakdown of outcomes, interventions and population groups reported in the reviews

Types of interventions	Social care populations	Quality of life outcomes (*n* = 34)				Prevention related outcomes (*n* = 25)		Other outcomes (*n* = 5)	
		Quality of life (*n* = 21)	Activities of Daily Living (*n* = 20)	Social participation (*n* = 9)	Dignity and control (*n* = 1)	Illness and/or events (*n* = 24)	Delaying need for services (*n* = 4)	Satisfaction (*n* = 4)	Safeguarding (*n* = 1)
Physical activity: *n* = 18		*n* = 11	*n* = 6	*n* = 1	*n* = 1	*n* = 9			
	People with LTC	√	√			√			
	Older people		√		√	√			
	People with MHP	√	√	√					
	People with learning disabilities	√							
Occupational therapy: *n* = 6		*n* = 4	*n* = 6	*n* = 1		*n* = 3	*n* = 1	*n* = 1	
	People with LTC	√	√			√	√	√	
	People with MHP	√	√	√		√			
Lay peer support: *n* = 3			*n* = 1			*n* = 3	*n* = 1		
	People with LTC		√	√		√	√		
Supported employment: *n* = 3		*n* = 1	*n* = 1	*n* = 3					
	People with MHP	√	√	√					
Alternative therapies: *n* = 2		*n* = 2	*n* = 1						
	People with LTC	√	√						
Assistive devices: *n* = 2									
	People with LTC								
Home‐Hazard Assessments: *n* = 2						*n* = 2			
	Older people					√			
Personal assistance: *n* = 2		*n* = 2	*n* = 2	*n* = 2		*n* = 2	*n* = 2	*n* = 2	
	People with PD and LD	√	√	√		√	√	√	
	Older people	√	√	√		√	√	√	
Safeguarding education: *n* = 1									*n* = 1
	Older people								√
Hip protectors: *n* = 1						*n* = 1			
	Older people					√			
Music therapy: *n* = 1				*n* = 1					
	People with LTC			√					
Case management: *n* = 1		*n* = 1							
	People with LTC	√							
Social support: *n* = 1						*n* = 1			
	People with LTC					√			
Structured communication: *n* = 1				*n* = 1					
	People with LTC			√					

LTC, long‐term conditions; MHP, mental health problems, PD, physical disabilities; LD, Learning disabilities

The reviews examining quality of life outcomes were most likely to measure general or overall quality of life (*n* = 21), followed by measures of people's ability to participate in activities of daily living (*n* = 20). A smaller number of reviews examined social participation (*n* = 9) and only one review measured outcomes relevant to dignity and control. The 25 reviews containing evidence on prevention‐related outcomes focused on reducing depression and poor mental health (*n* = 19), falls among older people (*n* = 5) and on preventing and reducing the need for health and social care services (*n* = 4).

Fourteen different types of interventions were investigated across the 43 reviews. Over two‐fifths of those (*n* = 18) focused on engagement in physical activity. The second most commonly reviewed intervention type was occupational therapy (*n* = 6). The remaining interventions were examined in three or fewer reviews. The population focus was primarily people with long‐term conditions (*n* = 27) such as cancer or Alzheimer's. Fewer reviews focused on older people (*n* = 9) or people with mental health problems (*n* = 5) and only three reviews focused on people with learning and/or physical disabilities.

Important gaps are revealed in the clustering and uneven distribution of review foci. For example, review‐level evidence on satisfaction with services and safeguarding is severely lacking and very few reviews cover those typically regarded as social care services. In terms of social care populations, evidence regarding people with physical or learning disabilities is also severely limited relative to that on older people and those with chronic conditions.

### Findings

Narrative syntheses of the evidence are presented by ASCOF outcomes and type of interventions. For each intervention/outcome combination, a summary statement is provided describing the direction of the findings and the strength of the evidence, indicated by the number of reviews, number of contributing studies, RCTs and nRCTs and whether meta‐analysis was undertaken. (A pooled estimate from a statistical meta‐analysis benefits from greater statistical power to detect an effect than an individual study, and therefore offers a more precise picture of the direction and size of the evidence.)

First we present a broad summary of the evidence (Table 3) which indicates that some social care interventions can have a positive impact on ASCOF outcomes. These include physical activity, supported employment/education, Tai Chi and personal assistance. The most consistent evidence is found for physical activity programmes for improving quality of life, dignity and control and preventing depression and mental health‐related illnesses. Overall, the evidence does not indicate that providing social care services causes harm except for in two reviews which contained evidence that interventions shown to be effective for some populations could potentially have a negative impact on vulnerable social care recipients. These included Tai Chi; although effective for older people in general, it was found to increase the rate of falls among frail older people. In addition, physical activity, which was found to have positive impacts on people exercising for rehabilitation after a period of ill‐health, was also found to have a negative impact on people exercising to support the management of their condition. This evidence makes clear the need for particular vigilance when implementing social care interventions among vulnerable groups. However, many of the social care interventions identified were either not shown to be effective or there is inconsistent and/or limited evidence on their effectiveness. For these interventions, it is not possible to give an indication as to whether or not they are likely to be effective in improving ASCOF outcomes.

**Table 3:** Summary of the evidence on the effectiveness of different social care interventions

Outcomes	Interventions measuring outcomes relevant to ASCOF			
	Conclusive evidence^a^		Inconclusive evidence	
	Evidence of positive effect	Not shown to be effective^b^	Inconsistent	Insufficient
*Enhancing overall quality of life*	**Physical activity** *Occupational therapy* *Personal assistance*	**Lay/Peer support** *Alternative therapies* *Case management*	*Supported employment*	
*Enabling activities of daily living*	**Occupational therapy** Physical activity *Assistive devices*	**Alternative therapies** *Case management*	*Supported employment* *Personal assistance*	*Lay/Peer support*
*Increasing social participation*	*Supportive employment/education* *Personal assistance*	**Structured communication**	Physical activity	*Music therapy* *Occupational therapy*
*Ensuring dignity and control*	*Physical activity*			
*Preventing depression & poor mental health*	**Physical activity** *Lay/Peer support*	*Social support* *Case management*	*Occupational therapy* *Alternative therapies*	*Occupational therapy* *Personal assistance*
*Preventing falls*	**Hip protectors** *Physical Activity (Tai Chi)* a *Home‐hazard assessment and modification*			
*Reducing the need for services*		*Lay/Peer support*		Personal assistance Occupational therapy
Satisfaction with Services	*Personal assistance*	*Case management*	Occupational therapy	
Safeguarding				Safeguarding training

a Stronger conclusive evidence is marked in bold. Tentative conclusions are in italics. Stronger evidence = corroborative evidence from multiple meta‐analyses or evidence from a single meta‐analysis with no conflicting evidence from narrative reviews. Tentative conclusions are drawn where evidence comes from narrative reviews only or where there is strong meta‐analytic evidence with limited conflicting evidence (i.e. a minority of evidence or narrative evidence).

b No evidence of difference between intervention and control group.

### Conclusive evidence

#### Evidence of positive effect for enhancing overall quality of life

##### Physical activity for improving overall quality of life (11 reviews; 75 RCTs)

Six of the eleven reviews found evidence that physical activity had a positive impact on the quality of life of people with a range of long‐term conditions (Gillison *et al*. 2009, Baillet *et al*. 2010, Hauser *et al*. 2010, Lin *et al*. 2011) and those with learning disabilities (Bartlo & Klein 2011). Five of these reviews were meta‐analyses totalling 45 studies. The meta‐analysis by Gillison *et al*. (2009) examined outcomes among two population groups; those exercising to manage their condition and those exercising for rehabilitation purposes. The positive findings relate to those in the management group only, with no evidence of difference found for the rehabilitation group. The remaining five reviews reported inconclusive findings. Three reviews on people with long‐term conditions (Lee *et al*. 2007, Lowe *et al*. 2009, Bradt *et al*. 2011) were inconclusive because of a lack of available evidence. Evidence in two reviews, one on people with long‐term conditions (Hall *et al*. 2009), and one on people with mental health problems (Schuch *et al*. 2011) was inconsistent. However, the majority of reviews and in particular those employing meta‐analysis and involving greater numbers of studies suggest that physical activity improves general quality of life, particularly for people living with chronic conditions.

##### Occupational therapy (OT) for improving overall quality of life (3 reviews; 8 RCTs)

One narrative review examining interventions to modify activity demands in self‐care activities for people with Alzheimer's found positive findings from four RCTs (Padilla 2011). Insufficient evidence in the remaining two narrative reviews meant conclusions could not be drawn about life skills programmes for people with mental health problems (Tungpunkom *et al*. 2012) and a range of occupational therapy programmes for people with Parkinson's disease (Dixon *et al*. 2007). Although, the overall evidence is limited, the positive findings from the single review contain sufficient evidence to suggests occupational therapy is beneficial for people with Alzheimer's and further research with other groups is warranted.

##### Personal assistance for improving overall quality of life (2 reviews; 1 RCT and 1 nRCT)

Two narrative reviews examined the effects on life satisfaction of personal assistance interventions – individualised support for people living in the community, delivered by a paid assistant – for people with physical and intellectual disabilities (Mayo‐Wilson *et al*. 2008) and older people (Montgomery *et al*. 2008). Evidence, which was drawn from the same two studies for each review, suggests a positive impact for both populations. Due to the limited pool of studies, however, this conclusion should be seen as tentative and further evidence should be sought.

#### Evidence not shown to be effective for enhancing overall quality of life

##### Alternative therapies for improving overall quality of life (2 reviews; 8 RCTs and 3 nRCTs)

A meta‐analytic review found no evidence of difference in quality of life for people recovering from stroke compared to controls receiving ‘sham’ acupuncture (Kong Jae *et al*. 2010). Jain and Mills (2010) narrative review on the impact of biofield therapies – a range of techniques that use subtle energy to stimulate the body's own healing process reported inconsistent evidence; finding positive effects for those living with chronic pain but conflicting evidence for cancer populations.

##### Lay/peer support for improving overall quality of life (1 review; 3 RCTs)

No evidence of difference in quality of life was found between people with long‐term chronic conditions receiving lay‐led self‐management programmes and those receiving usual care in a meta‐analytic review (Foster *et al*. 2007). Thus, further research is needed to corroborate this tentative finding.

##### Case management for improving overall quality of life (1 review; 4 RCTs)

Case management for people after they had suffered from a stroke was not shown to be effective for overall quality of life; none of the studies in this narrative review found significant differences between those receiving post‐stroke case management and control groups receiving usual care (Allison *et al*. 2011). Additional evidence and meta‐analysis should be pursued to confirm this finding.

#### Evidence of positive effect for enabling activities of daily living (ADL)

##### Occupational therapy for improving ADL (6 reviews; 26 RCTs)

A positive impact on ADL outcomes among people with long‐term conditions was found in two meta‐analytic reviews involving a total of 14 RCTs (Legg *et al*. 2007, Olazaran *et al*. 2010). Evidence in one narrative review (Padilla 2011) was conflicting; two of four included RCTs showed between‐group differences, while the other two did not. Evidence was insufficient in the remaining three reviews; two on people with long‐term conditions (Dixon *et al*. 2007, Hand *et al*. 2011) and one on individuals with mental health problems, Schizophrenia (Tungpunkom *et al*. 2012). The stronger meta‐analytic evidence suggests positive effects of occupational therapy. However, research is needed to establish its impact beyond populations with long‐term conditions. Moreover, the diversity of occupational therapy interventions within the included reviews indicates that further review‐level evidence about specific types of occupational therapy is needed.

##### Physical activity for improving ADL (8 reviews; 72 RCTs)

Four reviews contained insufficient evidence to draw a conclusion about the impact of physical activity on ADL outcomes; the reviews focused on older people (Forbes *et al*. 2008), people with arthritis (Lee *et al*. 2007), people with cancer (Lowe *et al*. 2009) and people with mental health problems (Schuch *et al*. 2011). Findings were conflicting or inconsistent within each of the remaining four reviews on older people (Daniels *et al*. 2008, Forster *et al*. 2009) people with osteoarthritis (Hall *et al*. 2009) and people with long‐term conditions (Gillison *et al*. 2009). However, strong meta‐analytic evidence in two of these reviews (Gillison *et al*. 2009, Hall *et al*. 2009) showed positive impacts. The Gillison *et al*. (2009) review found no evidence of difference between those exercising for rehabilitation purposes and controls regarding ADL outcomes, but pooled evidence from 13 studies on people exercising for condition management showed a benefit. In addition, the stronger evidence in the Hall *et al*. (2009) review showed positive outcomes; pooled evidence from four studies showed a benefit for self‐reported ADL, while a finding of no evidence of difference on another ADL outcome was based on a narrative review of just two studies. Some strong meta‐analytic evidence suggests physical activity may be beneficial for supporting ADL among some people with chronic conditions. However, further evidence is needed to clarify its potential among wider social care populations.

##### Assistive devices for improving ADL (2 reviews; 3 RCTs)

A limited pool of positive evidence indicates that further investigation of assistive devices is warranted. One narrative review found improvements in daily functioning for people with Alzheimer's disease due to modifications to the home setting (such as labels on drawers and visible emergency telephone numbers) and adaptive devices (such as pill reminder boxes) (Padilla 2011). A second review found insufficient evidence to draw a conclusion about assistive eye‐drop devices, though the single RCT had positive findings (Tuntland *et al*. 2009). Although the findings regarding assistive devices for improving ADL were consistently positive, the limited pool of studies suggests we should see the evidence as holding, overall.

#### Evidence not shown to be effective for enabling activities of daily living

##### Alternative therapies for improving ADL (1 review; 5 RCTs)

Acupuncture for improving ADL is not supported by evidence. A meta‐analysis on the impact of acupuncture on ADL outcomes for people recovering from stroke showed no evidence of difference between acupuncture and ‘sham’ acupuncture (Kong Jae *et al*. 2010).

##### Case management for improving ADL (1 review; 4 RCTs)

No evidence of difference in ADL outcomes was found between people receiving post‐stroke case management and controls receiving usual care in a narrative review (Allison *et al*. 2011).

#### Evidence of positive effect for increasing social participation

##### Supported employment and/or education for increasing social participation (3 reviews; 16 RCTs and 2 nRCTs)

Two reviews reported positive results. One meta‐analysed 11 RCTs and found that ‘Individual Placement Support’ – personalised interventions which integrate mental health and employment services – is effective for supporting social participation through employment (Bond *et al*. 2008). A second narratively reviewed two RCTs finding that supported education at the post‐secondary level for people with serious mental health problems is effective for increasing engagement in schooling (Arbesman & Logsdon 2011). A third review examined two types of programme finding no evidence of difference in the social participation of participants in a ‘community‐based vocational training and employment support’ programme compared to controls and inconsistent evidence for the effectiveness of a combined programme of ‘voluntary work and education’ (Dickson & Gough 2008). Overall, the majority of available evidence indicates that social participation among people with mental health problems can be improved by supporting people's engagement in employment and educational initiatives.

##### Personal assistance for increasing social participation (2 reviews; 1 RCT and 1 nRCT)

Two narrative reviews identified the same two studies, one RCT and one nRCT on the impact of personal assistance for people with physical and learning disabilities (Mayo‐Wilson *et al*. 2008) and for older people living in the community (Montgomery *et al*. 2008). Both reviews make cautious conclusion about the positive impact of personal assistance on social participation, judging the evidence to be limited due to the lack of studies for each population group.

#### Evidence not shown to be effective for increasing social participation

##### Structured communication for increasing social participation (1 review; 7 RCTs and 3 nRCTs)

Structured communication appears to be of little benefit for people with dementia in relation to social participation. Eight of ten included studies showed no evidence of difference between the intervention group and controls; a statistical meta‐analysis pooling evidence from five studies corroborated this finding (Vasse *et al*. 2010).

#### Evidence of positive effect for ensuring dignity and control

##### Physical activity for ensuring dignity and control (1 review; 6 RCTs)

A narrative review explored the effectiveness of Tai Chi as a single intervention and when combined with exercises, to support a reduction in the fear of falling among community and institution‐based older adults (Harling & Simpson 2008). The intervention was found to reduce fear in five of the six included trials. Although further research would be welcome, these findings support a tentative conclusion that Tai Chi has benefits for improving a sense of dignity and control among older people.

#### Evidence of positive effect for preventing depression and poor mental health

##### Physical activity for preventing depression and poor mental health (7 reviews, 39 RCTs)

Just over half of the reviews (*n* = 4), three of which were meta‐analyses, reported positive effects. The remaining three reviews contained insufficient evidence for a conclusion to be drawn. Participants in all reviews had long‐term conditions. The four reviews reporting positive evidence of impact found that preventing depression and poor mental health is possible through the application of a range of physical activity types for people with arthritis (Yohannes & Caton 2010), through aerobic exercise for people diagnosed with chronic pain (Hauser *et al*. 2010) or HIV (O'Brien *et al*. 2010) and for people with cancer who participated in yoga classes (Lin *et al*. 2011). A lack of evidence was found for the effectiveness of physical activity on depression in older adults with dementia (Forbes *et al*. 2008), Tai Chi for people with arthritis (Lee *et al*. 2007) and dance therapy for cancer patients (Bradt *et al*. 2011). The evidence thus appears fairly conclusive that physical activity can prevent or reduce poor mental health among people with long‐term conditions.

##### Lay/peer support for preventing depression and poor mental health (2 reviews, 12 RCTs)

One meta‐analytic review found a positive impact of lay‐led self‐management education on depression and anxiety for people with chronic conditions (Foster *et al*. 2007). One further narrative review found there was no evidence of a difference between group peer support and comparison experiences among people living with cancer (Hoey *et al*. 2008). Although the evidence base is not entirely clear cut, strong meta‐analytic evidence from the Foster *et al*. (2007) review indicates that peer support for people with LTCs may prevent depression and anxiety.

#### Evidence not shown to be effective for preventing depression and poor mental health

##### Social support for preventing depression and poor mental health (1 review, 8 RCTs)

This narrative review of a social support programme for people who have had a stroke found no evidence of impact for depression, distress or mood status (Salter *et al*. 2010). The lack of statistical meta‐analysis hampers our ability to draw definitive conclusions; however, the evidence as it stands does not provide grounds for recommending post‐stroke social support.

##### Case management for preventing depression and poor mental health (1 review, 6 RCTs)

This narrative review of primary care‐based follow‐up with a social care element for people who were recovering from stroke found no evidence of a difference in effect on mood between this intervention and usual care (Allison *et al*. 2011). While the evidence base does not currently support a recommendation of post‐stroke case management, further evidence and statistical meta‐analysis would provide greater clarity.

#### Evidence of positive effect for preventing falls

##### Hip protectors for preventing falls (1 review, 5 RCTs)

This meta‐analytic review evaluated the effects of double‐sided, hard‐shell hip protectors on hip fractures in older people living in nursing homes (Sawka *et al*. 2010). The review found that hip protectors reduced hip fractures, when compared with usual care. Although evidence is not available from multiple reviews, it seems reasonable to conclude that hip protectors are effective for preventing hip fracture among older people.

##### Physical activity (Tai Chi) for preventing falls (2 reviews, 11 RCTs)

Findings from these reviews were mixed. One meta‐analytic review concluded that Tai Chi is effective when compared to no exercise for reducing falls among healthy older adults but may be harmful for frail older adults (Leung *et al*. 2011). One further, narrative review concluded that there was weak evidence supporting the effectiveness of Tai Chi (as a single intervention, or combined with exercise) in reducing the number of falls in older adults (Harling & Simpson 2008). Overall, there is adequate evidence in the two reviews to suggest that Tai Chi is effective for reducing falls among healthy older adults. However, further evidence about the potential risks to frail older adults is required.

##### Home‐hazard assessment and modification for preventing falls (2 reviews, 5 RCTs)

These reviews focused on older adults living in the community and looked at practices such as the removal of rugs, installation of safety devices and behavioural counselling or education, e.g. advice on footwear or fall risks. The first, a meta‐analysis of three RCTs found this intervention was more effective than a control condition (Michael *et al*. 2010). The second, a narrative review, also found a significant positive impact on falls reduction (Costello & Edelstein 2008). However, the authors of the Costello review do not describe the quality of the included RCTs. The findings from this review should be regarded as tentative. As such, the evidence suggests that home‐hazard interventions may be effective, but more evidence is needed.

#### Evidence not shown to be effective for preventing the need for services

##### Lay/peer support for preventing the need for services (1 review, 9 RCTs)

This meta‐analytic review found no evidence of a difference in impact on the need for healthcare services when lay‐led self‐management programmes were compared with usual care for people with a range of long‐term conditions (Foster *et al*. 2007). Although evidence is not available from multiple reviews, the large number of studies contributing to a pooled statistical finding suggests the finding of no evidence of difference is robust.

#### Evidence of positive effect for improving satisfaction with services

##### Personal assistance for satisfaction with services (2 reviews, 1 RCT and 3 nRCTs)

These reviews, containing four studies and one study respectively, found that receiving personal assistance improves satisfaction with services among older people (Montgomery *et al*. 2008), and may improve satisfaction among people with physical or learning disabilities (Mayo‐Wilson *et al*. 2008) when compared with other forms of care. Further research to corroborate these tentative but promising findings is warranted.

#### Evidence not shown to be effective for improving satisfaction with services

##### Case management for satisfaction with services (1 review, 4 RCTs)

This narrative review found no evidence of the impact of case management on general satisfaction when compared with usual services for people who had had a stroke (Allison *et al*. 2011). None of the four RCTs included in the review showed a significant difference between groups on overall satisfaction.

#### Inconclusive evidence

The findings on inconclusive evidence are presented in Table 4.

**Table 4:** Findings on inconclusive evidence

Outcomes	Inconclusive Evidence	
Enhancing overall quality of life	Inconsistent	Supported employment for improving overall quality of life (1 review; 3 RCTs and 2 nRCTs) *Two different types of supported employment for people with mental health problems* – mixed findings. Voluntary work combined with education showed a positive impact (2 RCTs). However, no evidence of difference was found between groups receiving community‐based vocational training and employment and those receiving usual care: 1 RCT, 2 nRCTs; Narrative review (Dickson & Gough 2008)
Enabling activities of daily living	Inconsistent	Supported employment for improving ADL (1 review; 5 trials: 1 RCT and 4 nRCTs) *Vocational training (VT) and Voluntary work and education (VWE) programmes for people with mental health problems* – mixed findings. Some studies reporting a positive impact while others found no evidence of difference between groups VT = 1 RCT, and 2 nRCTs; VWE = 2 nRCTs; narrative review (Dickson & Gough 2008)Personal assistance for improving ADL (2 reviews; 1 RCT and 2 nRCTs) *Personal assistance for people with physical and learning disabilities or older people in the community* – mixed findings. Evidence of a significant positive impact on reducing unmet needs for ADL was found in two studies in each review. However, for a second ADL outcome – functional status – evidence from a total of three studies did not show a positive impact. Narrative review (Mayo‐Wilson *et al*. 2008, Montgomery *et al*. 2008).
	Insufficient	Lay peer support for improving ADL (1 review; 1 RCT) *Internet‐based peer‐moderated self‐management people with arthritis* – insufficient evidence. Firm conclusions could not be drawn from a single study, despite finding a significant reduction in activity limitation Narrative review (Bender *et al*. 2011)
Increasing social participation	Inconsistent	Physical activity for increasing social participation (1 review; 4 RCTs) *Exercise programmes on improving social participation for people with clinical depression –* Mixed findings. Two studies found evidence of a positive impact and two did not. Narrative review (Schuch *et al*. 2011)
	Insufficient	Music therapy for increasing social participation (1 review; 1 nRCT) *Music therapy for people with dementia* – lack of evidence, based on single nRCT of unknown quality. Narrative review (Chatterton *et al*. 2010)Occupational therapy for increasing social participation (1 review; 1 RCT) *Life skills training for people with schizophrenia* – lack of evidence based on a single RCT judged to be of ‘very low’ quality. Narrative review (Tungpunkom *et al*. 2012)
Preventing depression and poor mental health	Inconsistent	Occupational therapy (OT) for preventing depression and poor mental health (3 reviews, 7 RCTs)*Multi‐component OT interventions for people with long‐term conditions* – mixed findings. Cognitive group stimulation‐based OT showed positive effects for people with Alzheimer's (3 RCTs; meta‐analysis, Olazaran 2010). However, no evidence of difference was found for OT focused on activities of daily living for people recovering from stroke (2 RCTs; meta‐analysis, Legg 2007) and the effects of community‐based OT (for various LTCs) is hampered by a lack of robust evidence to draw conclusions from 2 RCTs; Narrative review (Hand *et al*. 2011). Alternative therapies for preventing depression and poor mental health (1 review, 6 RCTs, 2 nRCTs) *Biofield therapies for people with long‐term conditions* experiencing pain – mixed findings. Half the studies reported positive effects (2 RCTs; 2 nRCTs) while the remaining half (4 RCTs) found no evidence no evidence of difference between those receiving the intervention and controls. Meta‐analysis (Jain & Mills 2010)
	Insufficient	Occupational therapy (OT) for preventing depression and poor mental health (3 reviews, 3 RCTs) *A range of OT interventions for people with LTCs or mental health problems* – lack of evidence on the impact of OT led behaviour management for people with Alzheimer's, 1 RCT; Meta‐analysis (Olazaran *et al*. 2010); life skills training for people with mental health conditions, 1 RCT; Narrative review (Tungpunkom *et al*. 2012) and stimulation therapy for dementia patients, 1 RCT, Narrative review (Padilla 2011) Personal assistance for preventing depression and poor mental health (1 review, 1 nRCT) *Individualised support (≥20 hours/week) for older people delivered by a paid assistance* – lack of evidence with only 1 nRCT identified. Narrative review (Montgomery *et al*. 2008)
Preventing falls	Insufficient	Personal assistance for preventing the need for services (2 reviews, 1 RCT) *Personal assistance for people with physical/intellectual disability or older people living in the community* ‐ lack of positive evidence that personal assistance reduces long‐term institutional care Narrative review (Mayo‐Wilson *et al*. 2008, Montgomery *et al*. 2008)
Reducing the need for services	Insufficient	Occupational therapy for preventing the need for services (1 review, 3 RCTs) *Institutional care in people who had suffered a stroke* – insufficient evidence found – particularly as the use of institutional care was difficult to disentangle from other measures (e.g. mortality). Narrative review (Legg *et al*. 2007)
Satisfaction with services		Occupational therapy for satisfaction with services (1 review, 2 RCTs) *OT focused on activities of daily living for people recovering from stroke* – insufficient evidence due to the small number of studies and incomplete reporting of data Narrative review (Legg *et al*. 2007)
Safeguarding	Insufficient	Safeguarding training *Safeguarding interventions for any social care population –* lack of conclusive review‐level evidence on safeguarding. Only 1 RCT, of unknown quality, was found in a review on the effects of an educational programme for nursing home staff. Narrative review (Lindbloom *et al*. 2007)

## Discussion

The main objective of the meta‐review was to provide a summary of the evidence on which social care interventions can effectively improve outcomes for services users. Through this process, we identified a number of gaps in the evidence that will be of concern to social care recipients as well as health and social care policy makers and professionals.

Despite the breadth of evidence contained within this meta‐review, the most striking gap is the lack of review‐level evidence on many of the adult social care interventions, outcomes and population groups we would expect to find. First, while there is a large body of reviewed evidence on physical activity and occupational therapy interventions, other types of social care interventions are only examined in one or two reviews and many social care interventions, relevant to the UK, such as assisted living, community transport and home help are not examined at all.

Second, the review‐level evidence on two of the ASCOF outcomes, satisfaction with services and safeguarding, is severely limited. The only evidence on safeguarding came from a single review, the findings of which were insufficient due to a lack of evidence. The significance of the lack of evidence on satisfaction outcomes should not be underestimated. While this meta‐review was firmly focused on outcomes, evidence on satisfaction provides important insights into whether such interventions are acceptable as well as effective. For example, while the included review by Sawka *et al*. (2010) indicates that hip protectors are effective for reducing fall related injuries among older people, other evidence (not includable in this meta‐review) indicates that people would not be satisfied with the provision of hip protectors as many find them uncomfortable to wear (Van Schoor *et al*. 2002, Gillespie *et al*. 2010). For many providers and recipients of social care, these types of issues will be just as salient as how effective interventions are. Satisfaction with services and safeguarding are key outcomes as set out in the ASCOF, and despite efforts to identify research on safeguarding interventions, it is clear that evidence on the effectiveness of interventions is under‐examined for these outcomes.

Third, evidence on interventions for people with long‐term conditions, although relevant, currently dominates the systematic review literature on effectiveness. Very little review‐level evidence on the effectiveness of social care interventions for people with physical or learning disabilities has been identified. It remains unclear whether the reason for this is because the primary research has not been conducted or because systematic reviews for these population groups have not yet been commissioned and produced. The latter seems more likely because we did not find any ‘empty reviews’ covering these populations.

We also found that the predominance of evidence in this meta‐review has some kind of a link to health. For example, physical activity interventions are provided to enhance health as much as they are quality of life. Although people with long‐term conditions may have social care needs, they will invariably also have healthcare needs and receive healthcare services. In terms of outcomes, the prevention outcomes examined nearly all relate to health events or health service use, while QoL measures can be very explicitly health‐focused, the Health‐related Quality of Life (HRQL) measurement tool being a key example. Moreover, the domains of quality of life predominantly examined in the reviews contained within this report are general QoL and ADL, while the less health‐focused aspects of social participation and dignity and control were measured far less often. Thus, the relevance to social care of the quality of life measures used in many of these reviews is questionable. Social care researchers point out that as social care has fundamentally different objectives to healthcare, different measures are needed that reflect the impact and value of social care interventions (Netten *et al*. 2012). This disconnect between the objectives of health‐related measures and the objectives of the social care interventions mean that some positive or even some harmful impacts may be missed.

A further reason for the high presence of health‐related interventions, populations and outcomes could also partly be because evaluation of the effectiveness of interventions is more common within the health literature and because the systematic review literature has a longer history in health than in social care (Oakley *et al*. 2005). The scarcity of trials in social care has been attributed to a lack of funding and training of skilled professionals to undertake such research and the challenging aspects of trial methodology for social care, such as establishing a viable control group, threats to internal validity when conducting research in highly variable contexts (MacDonald 1999) and the need to address ethical and service user issues, all of which require time, skills and resource. The social care literature has often been more focused on examining the processes involved in the delivery of complex social interventions, or the experience of care for users, rather than efficacy. If we had been tasked with answering research questions on implementation, we may have identified a greater and more representative spread of social care interventions. However, if data on implementation factors, such as feasibility, fidelity, and acceptability were collected in conjunction with trials, greater understanding of what contributes to effectiveness could possibly be obtained. The current dearth of experimental evidence on the effectiveness of social care interventions makes clear that while the need for evidence‐informed social care has long been recognised (MacDonald 2003), there is still a need to push this agenda further in terms of ensuring that complex social care interventions are subjected to rigorous evaluation and are subjected to systematic review.

The findings from this meta‐review provide direction for the commissioning of new primary research and further systematic reviews of primary evidence. In particular, the effectiveness of safeguarding interventions urgently needs addressing, especially given the recent failures of care for social care populations (DH 2011). A greater body of review‐level evidence on interventions more closely related to the role of social care workers is also required. For example, evidence on the kind of complex interventions routinely undertaken by social care workers and local authorities. As highlighted, the evidence base would also benefit from systematic reviews on the effectiveness of social care interventions for people with physical and learning disabilities and evidence regarding the efficacy of interventions falling under recent policy‐directed initiatives such as ‘personalisation’ (DH 2010b). Given the paucity of evidence found to date, satisfaction with services needs to be a routine outcome measured in evaluations of social care. A further priority is the need for further use and development of social care relevant outcomes such as social care related quality of life measures in research (Netten *et al*. 2012).

### Strengths and limitations

Summarising data from systematic reviews has enabled us to bring together crosscutting evidence on the efficacy of interventions relevant to social care. Although conducting a meta‐review provides a pragmatic approach to answering broad policy questions within a short‐timeframe (Thomas *et al*. 2013), there are nevertheless limitations. One of the main impositions of this method is that the evidence examined was limited to evaluations of social care interventions reported in systematic reviews. It is clear from the evidence assessed, that particular interventions, outcomes and populations are predominant within such review literature, much of which has a health rather than a social care focus.

A second limitation when utilising meta‐review methodology is the distance between the reviewers and the original studies. The lack of access to original study data means we were reliant on review authors’ reporting and interpretation of findings, constraining our efforts to interpret statistical effect sizes. We also had to take at face‐value authors’ quality assessments of their included studies. Their different approaches to critical appraisal, differing levels of description about study quality and differences between reviews in terms of what constituted an acceptable level of quality mean it was not possible to apply consistent inclusion criteria relating to the quality of included trials.

Our search strategy is limited to systematic reviews published in English before 2012. Thus, despite our best efforts, our search may not have captured reviews on the effectiveness of social care interventions from non‐English‐speaking OECD countries. Since our searches were carried out we are aware of only two potentially relevant reviews: on the effectiveness of personal budgets for people with mental health problems (Webber *et al*. 2014), and an update of an included review by Forbes *et al*. (2015) on exercise for people with dementia. Both reviews warrant consideration in any further updates on the evidence base for improving outcomes in adult social care.

A key issue we also faced when undertaking this meta‐review, spanning all of social care, was the extent to which we should ‘lump’ or ‘split’ review‐level findings when summarising the evidence (Weir *et al*. 2012). By grouping interventions, we were able to identify common findings within broad interventions types and assess the generalisability and consistency of those findings within each outcome domain for different social care populations. However, important differences that could contribute to explaining variation across findings might also have been concealed by those groupings. Future reviews would benefit from maintaining a broad scope to capture the complexity of social care interventions and explore heterogeneity where possible, either narratively or statistically via sub‐group analysis (Grimshaw *et al*. 2003).

Despite these limitations, this meta‐review was able to provide review‐level evidence from 43 systematic reviews containing approximately 300 individual studies and many thousands of participants, about the impact of interventions measuring adult social care outcomes. Synthesising this evidence contributes to the production of an accessible evidence base for policy makers and social care practitioners, as well as social care researchers. A second key strength of this review is that it not only illustrates which evaluated social care interventions are effective and which are not, but it also makes clear important evidence on potentially harmful interventions and explores how much impact social care interventions have on ASCOF outcomes.

## Conclusion

The greatest portion of evidence included in this meta‐review is about physical activity: evidence suggests that these types of interventions can be effective for people with long‐term conditions and non‐frail older people and may address both quality of life and prevention outcomes. Moreover, although physical activity interventions may typically be regarded as not within the remit of social care, they may be relatively cheap and easy to implement, and therefore worth considering. More complex, and perhaps more recognisably social care interventions need to be subject to evaluation, review and synthesis. The key message from this meta‐review is the need to recognise the influence of contextual factors on the success of social care interventions, in particular the need for safety measures when implementing social care interventions with particularly vulnerable groups.

## Disclaimer

The views expressed in the publication are those of the authors and not necessarily those of the Department of Health.
