# Supplementary material for: Gaps in the evidence on improving social care outcomes: findings from a meta‐review of systematic reviews
Source: Health Soc Care Community. 2015 Oct 26;25(4):1287–303. doi: 10.1111/hsc.12300 (PMC5484323; doi:10.1111/hsc.12300)
Supplement: Supplementary file 1 [file HSC-25-1287-s001.docx]

Supporting material

**Gaps in the evidence on improving social care outcomes: findings from a meta-review of systematic reviews to support the Adult Social Care Outcomes Framework**

Table of contents

[1 PRISMA checklist 2](#_Toc420573622)

[2 Methods 5](#_Toc420573623)

[2.1 Screening criteria for the in-depth review 5](#_Toc420573624)

[2.2 Quality and relevance screening for in-depth meta-review 8](#_Toc420573625)

[2.3 Search strategy 9](#_Toc420573626)

[2.4 Example search string for bibliographic database: IBSS 9](#_Toc420573627)

[2.5 Data extraction tool for meta-review synthesis 17](#_Toc420573628)

[3 Design and quality of systematic reviews 22](#_Toc420573629)

[3.1 Quality of life outcomes 22](#_Toc420573630)

[3.2 Prevention outcomes 28](#_Toc420573631)

[3.3 Satisfaction with services outcomes 34](#_Toc420573632)

[3.4 Safeguarding outcomes 35](#_Toc420573633)

# PRISMA checklist

| Section/topic | # | Checklist item | Reported on page # | |
| --- | --- | --- | --- | --- |
| TITLE | | |  | |
| Title | 1 | Identify the report as a systematic review, meta-analysis, or both. | #1 | |
| ABSTRACT | | |  | |
| Structured summary | 2 | Provide a structured summary including, as applicable: background; objectives; data sources; study eligibility criteria, participants, and interventions; study appraisal and synthesis methods; results; limitations; conclusions and implications of key findings; systematic review registration number. | #1 | |
| INTRODUCTION | | |  | |
| Rationale | 3 | Describe the rationale for the review in the context of what is already known. | #2 | |
| Objectives | 4 | Provide an explicit statement of questions being addressed with reference to participants, interventions, comparisons, outcomes, and study design (PICOS). | #2 | |
| METHODS | | |  | |
| Protocol and registration | 5 | Indicate if a review protocol exists, if and where it can be accessed (e.g., Web address), and, if available, provide registration information including registration number. | No – but full report available | |
| Eligibility criteria | 6 | Specify study characteristics (e.g., PICOS, length of follow-up) and report characteristics (e.g., years considered, language, publication status) used as criteria for eligibility, giving rationale. | #3 | |
| Information sources | 7 | Describe all information sources (e.g., databases with dates of coverage, contact with study authors to identify additional studies) in the search and date last searched. | #3 | |
| Search | 8 | Present full electronic search strategy for at least one database, including any limits used, such that it could be repeated. | Provided in web-appendix | |
| Study selection | 9 | State the process for selecting studies (i.e., screening, eligibility, included in systematic review, and, if applicable, included in the meta-analysis). | #3 | |
| Data collection process | 10 | Describe method of data extraction from reports (e.g., piloted forms, independently, in duplicate) and any processes for obtaining and confirming data from investigators. | #3; #4 | |
| Data items | 11 | List and define all variables for which data were sought (e.g., PICOS, funding sources) and any assumptions and simplifications made. | #3 | |
| Risk of bias in individual studies | 12 | Describe methods used for assessing risk of bias of individual studies (including specification of whether this was done at the study or outcome level), and how this information is to be used in any data synthesis. | Quality appraisal provided #3 | |
| Summary measures | 13 | State the principal summary measures (e.g., risk ratio, difference in means). | n/a | |
| Synthesis of results | 14 | Describe the methods of handling data and combining results of studies, if done, including measures of consistency (e.g., I^2^) for each meta-analysis. | Equivalent for meta-reviews on #3-4 and #10-11 | |
| Section/topic | # | Checklist item | Reported on page # | |
| Risk of bias across studies | 15 | Specify any assessment of risk of bias that may affect the cumulative evidence (e.g., publication bias, selective reporting within studies). | n/a: meta-review | |
| Additional analyses | 16 | Describe methods of additional analyses (e.g., sensitivity or subgroup analyses, meta-regression), if done, indicating which were pre-specified. | n/a: meta-review | |
| RESULTS | | | |  |
| Study selection | 17 | Give numbers of studies screened, assessed for eligibility, and included in the review, with reasons for exclusions at each stage, ideally with a flow diagram. | | #8 |
| Study characteristics | 18 | For each study, present characteristics for which data were extracted (e.g., study size, PICOS, follow-up period) and provide the citations. | | #8-9; more detail provided in main report |
| Risk of bias within studies | 19 | Present data on risk of bias of each study and, if available, any outcome level assessment (see item 12). | | n/a: meta-review |
| Results of individual studies | 20 | For all outcomes considered (benefits or harms), present, for each study: (a) simple summary data for each intervention group (b) effect estimates and confidence intervals, ideally with a forest plot. | | #12-19 as appropriate for a meta-review |
| Synthesis of results | 21 | Present results of each meta-analysis done, including confidence intervals and measures of consistency. | | n/a |
| Risk of bias across studies | 22 | Present results of any assessment of risk of bias across studies (see Item 15). | | n/a |
| Additional analysis | 23 | Give results of additional analyses, if done (e.g., sensitivity or subgroup analyses, meta-regression [see Item 16]). | | n/a |
| DISCUSSION | | | |  |
| Summary of evidence | 24 | Summarize the main findings including the strength of evidence for each main outcome; consider their relevance to key groups (e.g., healthcare providers, users, and policy makers). | | #11 12-19 |
| Limitations | 25 | Discuss limitations at study and outcome level (e.g., risk of bias), and at review-level (e.g., incomplete retrieval of identified research, reporting bias). | | #21 |
| Conclusions | 26 | Provide a general interpretation of the results in the context of other evidence, and implications for future research. | | #20-22 |
| FUNDING | | | |  |
| Funding | 27 | Describe sources of funding for the systematic review and other support (e.g., supply of data); role of funders for the systematic review. | | #22 |

# Methods

## Screening criteria for the in-depth review

| **Stage** | **Criterion*** | | **Rationale** | |
| --- | --- | --- | --- | --- |
|  | **Reviews must:** | | |  |
| A – Screen reviews for usability, currency and relevance | 1 | ** Be published in English | | The timescale of this review of evidence did not allow for translation of studies published in other languages |
|  | 2 | ** Be reported in or after 2000 | | This allowed the map to focus upon recent systematic reviews of research |
|  | 3 | ** Not focus exclusively on children and/or young people, or report on a mixed population with no findings specific to over 18s | | To identify systematic reviews that provide findings on the topic(s) of interest |
|  | 4 | ** Not be restricted to studies from non-OECD countries | | As the purpose of this meta-review is to inform UK practice, this criterion ensures a reasonable level of comparability with the modern and well-funded social care system in the UK |
|  | 5 | ** Report findings from social care populations | | To identify systematic reviews that provide findings for the population(s) of interest |
|  | 6 | ** Be a systematic review that describes a search strategy and criteria for including studies | | To ensure included systematic reviews have taken reasonable steps to minimise bias |
|  | 7 | ** Focus on social care services  (Note: Providers did not need to be specified for an intervention to be included, but reference to certain job titles (specifically doctor, nurse, physiotherapist, psychotherapist) was taken to indicate that a service was not primarily a social care service.) | | To identify systematic reviews that provide findings on the topic(s) of interest |
|  | 8 | ** Examine the effects of interventions | | Some systematic reviews exist that address different kinds of research question, for example user and provider perspectives on services however the focus of this review is on effectiveness |
|  | 9 | ** Measure one or more of the ASCOF outcomes (quality of life, prevention, satisfaction, safeguarding) | | To identify systematic reviews that provide findings on the topic(s) of interest |
|  | 10 | ** Not have a limited social care focus (i.e. reviews were excluded if they: i) examined a range of interventions that included social care interventions, without providing summary statements specific to social care interventions; or ii) solely examined multi-disciplinary interventions, unless these were reported as led by social workers or occupational therapists) | | To identify systematic reviews that provide findings on the topic(s) of interest |
|  | 11 | ** If focused on older people, not use an inclusion criterion of age < 65 | | To identify systematic reviews that provide findings for the population(s) of interest |
|  | 12 | ** Report usable summary statements of findings relevant to ASCOF outcomes | | The timescale of this systematic review did not allow for a synthesis of findings reported on a study by study basis, but required that review authors had already synthesised individual study findings, either in a narrative or a numerical form |
| Additional substantive criterion for in-depth review |  | Be reported in or after 2007 | | This allowed the systematic review to focus upon the most recent reviews of research. It assumes that earlier reviews are likely to have been published after 2006 in an updated form |
| Additional substantive criterion for in-depth review |  | Report findings from social care populations other than carers | | During the course of the systematic map, an existing systematic review of systematic reviews was identified on interventions to support carers |
| B – Screen reviews for review quality |  | Use a comprehensive search strategy involving two or more electronic databases | | To ensure inclusion of comprehensive reviews |
|  |  | Explicitly describe the inclusion criteria applied to studies in the review and present these as part of a report’s methods section | | To ensure that reviews are systematic rather than selective, i.e. to remove any ambiguity about the scope of included reviews so it is clear what evidence they contribute to this review |
| C – Screen reviews for usable data |  | Provide one or more summary statements that were produced exclusively from studies with a controlled trial design | | To ensure evidence used to identify effective interventions is reasonably trustworthy  (findings used for Chapter 3 and 4) |
|  |  | Conduct a meta-analysis and provide details of the size of effects | | To enable reviewers to interpret evidence about the scale of impact of interventions  (findings used for Chapter 5) |

* For definitions of the concepts used in these criteria, see main paper
** Indicates that this was one of the initial criteria used to produce a systematic map

## Quality and relevance screening for in-depth meta-review

When moving from the systematic map, the exclusion criteria was operationalised into the following screening tool and studies were re-screened for inclusion.

| Questions |  |
| --- | --- |
| 1. SEARCH – review searches two or more databases? | - 1. **Yes** – details   2. **No** – details |
| 1. INC CRITERIA- review has explicit inclusion criteria? | - 1. **Yes** – details   2. **No** – details |
| 1. QUALITY – review includes randomised control trials (RCTs) /controlled trials (CTs) only - or summary statements = RCT/CT only | - 1. **Yes** - RCTs/CTs ONLY - details   2. **Yes** - there are summary on RCT/CT evidence only – details   3. **No** – details |
| 1. PROVIDER - does review provide evidence about social care led interventions? | - 1. **Yes** - exclusively social care led (details)   2. **Yes** - summary statements soc care led (details)   3. **No** (details) *Use this code if: -* *- the review does not specify who provider is and it's not implicitly clear that it would be a social care led intervention* *OR* *- if it includes some social care led interventions - but no summary statements about them specifically* |
| 1. OUTCOMES - does review provide ASCOF outcome SS? | - 1. **Yes** – details   2. **No** – details |
| 1. FOR INDEPTH REVIEW? | - 1. Include - meets all criteria   2. Exclude - one or more quality and relevance criteria not met   3. Exclude - not social care provider   4. Exclude - outcomes - no relevant outcomes measured |

## Search strategy

### Developing search terms

A comprehensive search strategy was developed in consultation with an information specialist with extensive experience of conducting searches for systematic reviews, and with a social care researcher from Social Care Institute of Excellence (SCIE). Thesaurus terms were used to capture various concepts, which were combined in the following search string: (social care services OR social care outcomes) AND (social care populations) AND (review). Where no thesaurus term existed for a concept, free-text terms were used in the title and abstract field. Date restrictions were employed on some databases. Searches were carried out between 01/02/12 and 20/02/12. The reference lists of all reviews included in the map were screened for further potentially includable reviews. One of the search strategies employed is presented in 2.3 (below) to illustrate the extensive and comprehensive nature of the searches. Full details of the review’s searches are available on request

### Search sources

The following bibliographic databases were searched for pertinent systematic reviews:

- The Cochrane Library
- Database of Abstracts of Reviews of Effects (DARE)
- Health Technology Assessment (HTA)
- National Health Service Economic Evaluation Database (NHS EED)
- PubMed
- Embase
- PsycInfo
- ASSIA
- Social Science Citation Index
- IBSS
- Sociological Abstracts and Social Services Abstracts
- Social Care Online

These database searches were supplemented with searches by hand of:

- The website of the Social Policy Research Unit, University of York, <http://php.york.ac.uk/inst/spru/pubs/adult.php>
- The Journal of Elder Abuse and Neglect (2008-2011).

## Example search string for bibliographic database: IBSS

FINAL IBSS search. Results: 264

Platform: CSA. Search run 1 February 2011 by Rebecca Rees, date range: 2000-2012

[Annotated to identify different conceptual areas]

[Controlled term search for social care interventions and outcomes] ((((DE="**occupational therapy**") or((DE="**social work**") or(DE="**social workers**") or(DE=("**community care**" or "**social security**" or "**social services**" or "**social support**")) or(DE="**social services**")) or((DE="**residential care**") or(DE="**care of the aged**"))) or(DE="**community services**") or(DE="Long-term **care**") or(DE="**Benefit plans**" OR DE="**Payments**")) or((DE=("**Social integration**" or "**Social exclusion**" or "**Empowerment**” or "**Social participation**" or "**Autonomy**" or "**Decision making**" or "**Quality of life**")) or(DE=("**employment**" or "**access to employment**" or "**employment opportunities**" or "**employment situation**" or "**full time employment**" or "**part time employment**" or "**temporary employment**")) or(DE=("**Resident satisfaction**" or "**Satisfaction**" or "**Information acquisition**")) or(DE=**Prevention** AND DE=**Hospitalization**) or(DE="**sexual abuse**" or DE="**abuse of the aged**" or DE="**domestic violence**" or DE= "**sexual assault**" or DE="**human rights**" or DE="**injuries**")))

And

[Controlled term search for social care populations] (((DE="**aged**") or(DE=("**Alzheimer’s disease**" or "**dementia**")) or(DE=("**ageing**" or "**senescence**"))) or((DE=("**Mental illness**" or "**Schizophrenia**" or "**Social psychiatry**" or "**Mentally disabled**" or "**Psychoses**" or "**Addiction**" or "**Alcoholism**" or "**Trauma**" or "**Psychosis**" or "**Social psychiatry**" or "**Depression**" or "**Substance use**" or "**Drug use**" or "**Anorexia nervosa**" or "**Eating disorders**" or "**Personality disorders**")) or(DE="**mental health**")) or(DE=("**Disabled persons**" or "**Disability**" or "**Blindness**" or "**Deafness**")) or(DE=("**Learning disabilities**" or "**Mentally disabled**")) or(DE=("**caring**")))

And

[Controlled term search for systematic reviews] ((DE="**Review articles**") or(((KW=**systematic** within 2 **review***) or(((KW=**inclusion** within 5 **criteri***) or(KW=("**systematic***" or "**critical**" or "**study selection**" or "**predetermined**" or "**exclusion criteri***" or "**main outcome measures**")) or(KW=**standard** within 2 **care**)) and(KW=("**survey***" OR "**overview***" OR "**review***" OR "**search***" OR "**handsearch***" OR "**analysis**" OR "**critique**" OR "**appraisal**")) and((KW=**clinical** within 3 **studies**) or(KW=("**survey***" OR "**overview***" OR "**review***" OR "**search***" OR "**handsearch***" OR "**analysis**" OR "**critique**" OR "**appraisal**")) or((KW=**clinical** within 3 **studies**) or(KW="**literature**" OR "**articles**" OR "**publication***" OR "**bibliographies**" OR "**published**" OR "**unpublished**" OR "**citation***" OR "**database**" or "**internet**" OR "**textbooks**" OR "**scales**" OR "**papers**" OR "**datasets**" OR "**trials**" OR "meta-analy*" OR "**intervention***" OR "**treatment outcome***"))))) or(KW=("**evidence based**" OR "**best practice***" OR "**evidence synthesis**"))))

OR

[Free-text search for social care interventions and outcomes] ((**TI**= ("**Social care**" OR "**social service**" OR "**social services**" OR "**adult service**" OR "**adult services**" OR "**social work service***" OR "**social support service***" OR "**social care service***" OR "**social care support**" OR "**home care service***" OR "**home caring service***" OR "**homecare service***" OR "**social intervention***" OR "**welfare service***" OR "**welfare recipient***" OR "**social welfare**" OR "**social program***" OR "**adult care service***" OR "**personal care**" OR "**community care**" OR "**community services**" OR "**community mental health team***" OR "**community mental health care**" OR "**community mental healthcare**" OR (**community** within 2 ("**day**" OR "**housing**" OR **volunteer** OR **social** OR **support**)) OR "**community based care**" OR "**case management**" OR "**sheltered work***" OR "**fountain house***" OR "**fountain-house***" OR "**clubhouse***" OR "club-house*" OR "**transitional employ***" OR "**housing**" OR "**domiciliary care**" OR "**domiciliary assistan***" OR "**non institutional care**" OR "**non resident care**" OR "**residential care**" OR "**home-based care**" OR "**home based care**" OR "**home health care**" OR "**home healthcare**" OR "**home assistance**" OR "**homecare**" OR "**restorative home care**" OR "**domestic care**" OR "**outreach services**" OR "**Outreach program***" OR "**Assertive outreach**" OR "**Peer outreach**" OR "**Home help**" OR "**meal service***" OR (**meals** within 2 **wheels**) OR "**Meal delivery**" OR "**Meal program***" OR "**Meal distribution**" OR "**community outreach**" OR "**Volunteer outreach**" OR "**Day service***" OR "**Assisted care**" OR "**Home visiting**" OR "**residential care**" OR "**older peoples home***" OR "**Respite care**" OR "**nursing home**" OR "**care home***" OR "**Residential home***" OR "**Daycare centres***" OR "**Nursing homes**" OR "**therapeutic communities**" OR "**assertive community treatment**" OR "**befriending**" OR "**Peer support**" OR "**Peer network***" OR "**Help group***" OR "**Help network***" OR "**Support group***" OR "**Support network***" OR "**Supportive services**" OR "**Mutual support**" OR "**Community support**" OR "**care** co-ordinator*" OR "**care coordinator***" OR "**Social worker***" OR "**Care supervi?or***" OR "**Care** worker/s" OR "**Care Facilitator***" OR "**Care adviser***" OR "**Care advizer***" OR "**Case manager***" OR "**Outreach worker***" OR "**Care assistant***" OR "**Health aide***" OR "**homemaker service***" OR "**Professional carer***" OR "**personal assistant***" OR "**personal assistance**" OR "**support worker***" OR "**consumer directed care**" OR "**flexible funding**" OR "**self directed care**" OR "**self directed support**" OR "**self managed care**" OR "**self managed support**" OR "**user directed care**" OR "**user directed support**" OR "**Consumer directed support**" OR "**direct payment**" OR "**direct payments**" OR "**person centred planning**" OR "**person centered planning**" OR "**person centred support**" OR "**person centered support**" OR "**user centred support**" OR "**user centred planning**" OR (**cash** within 2 **care**) OR (**cash** within 2 **counseling**) OR (**cash** within 2 **counselling**) OR "**individual budgets** " OR "**cash assistance**" OR "**attendance allowance**" OR "**Welfare benefit***" OR "**Welfare system***" OR "**Income support**" OR "**Benefit payment***" OR "**Social securit***" OR "**Mobility allowance***" OR "**Sickness benefit***" OR "**Invalidity benefit***" OR "**Disability benefit***" OR "**Respite care**" OR "**integrated service***" OR "**Services integration**" OR "**Social planning**" OR "**Reablement**" OR "re-ablement" OR "Re-enablement" OR "**Response Services**" OR "**Continuing Care**" OR "**transitional services**" OR "**Rehabilitation**" OR "**telerehabilitation**" OR "**electronic aids**" OR ("**electronic devices**" near **living**) OR (**aids** near "**daily living**") OR "**Assistive devices**" OR "**smart home***" OR "**Smart hous***" OR "**Smart technolog***" OR (**robot*** near **assist***) OR (**home** near **telecare**) OR (**home** near **telepresence**) OR "**befriending**" OR "**mentoring**" OR (**Control** within 2 "**daily life**") OR "**Independent living**" OR "**Living independently**" OR "**promote independence**" OR (**living** within 2 **home**) OR "**employment**" OR "**Live independently**" OR "**Independently live**" OR "**Independent lives**" OR "**Assisted living**" OR "**Assisted home***" OR "**Supported living**" OR "**social assistance**" OR "**socially assistive**" OR "**Promoting independence**" OR "**Carer consultation***" OR "**Person centred approach***" OR "**Person centred planning**")) or(AB= ("**Social care**" OR "**social service**" OR "**social services**" OR "**adult service**" OR "**adult services**" OR "**social work service***" OR "**social support service***" OR "**social care service***" OR "**social care support**" OR "**home care service***" OR "**home caring service***" OR "**homecare service***" OR "**social intervention***" OR "**welfare service***" OR "**welfare recipient***" OR "**social welfare**" OR "**social program***" OR "**adult care service***" OR "**personal care**" OR "**community care**" OR "**community services**" OR "**community mental health team***" OR "**community mental health care**" OR "**community mental healthcare**" OR (**community** within 2 ("**day**" OR "**housing**" OR **volunteer** OR **social** OR **support**)) OR "**community based care**" OR "**case management**" OR "**sheltered work***" OR "**fountain house***" OR "**fountain-house***" OR "**clubhouse***" OR "club-house*" OR "**transitional employ***" OR "**housing**" OR "**domiciliary care**" OR "**domiciliary assistan***" OR "**non institutional care**" OR "**non resident care**" OR "**residential care**" OR "**home-based care**" OR "**home based care**" OR "**home health care**" OR "**home healthcare**" OR "**home assistance**" OR "**homecare**" OR "**restorative home care**" OR "**domestic care**" OR "**outreach services**" OR "**Outreach program***" OR "**Assertive outreach**" OR "**Peer outreach**" OR "**Home help**" OR "**meal service***" OR (**meals** within 2 **wheels**) OR "**Meal delivery**" OR "**Meal program***" OR "**Meal distribution**" OR "**community outreach**" OR "**Volunteer outreach**" OR "**Day service***" OR "**Assisted care**" OR "**Home visiting**" OR "**residential care**" OR "**older peoples home***" OR "**Respite care**" OR "**nursing home**" OR "**care home***" OR "**Residential home***" OR "**Daycare centres***" OR "**Nursing homes**" OR "**therapeutic communities**" OR "**assertive community treatment**" OR "**befriending**" OR "**Peer support**" OR "**Peer network***" OR "**Help group***" OR "**Help network***" OR "**Support group***" OR "**Support network***" OR "**Supportive services**" OR "**Mutual support**" OR "**Community support**" OR "**care** co-ordinator*" OR "**care coordinator***" OR "**Social worker***" OR "**Care supervi?or***" OR "**Care** worker/s" OR "**Care Facilitator***" OR "**Care adviser***" OR "**Care advizer***" OR "**Case manager***" OR "**Outreach worker***" OR "**Care assistant***" OR "**Health aide***" OR "**homemaker service***" OR "**Professional carer***" OR "**personal assistant***" OR "**personal assistance**" OR "**support worker***" OR "**consumer directed care**" OR "**flexible funding**" OR "**self directed care**" OR "**self directed support**" OR "**self managed care**" OR "**self managed support**" OR "**user directed care**" OR "**user directed support**" OR "**Consumer directed support**" OR "**direct payment**" OR "**direct payments**" OR "**person centred planning**" OR "**person centered planning**" OR "**person centred support**" OR "**person centered support**" OR "**user centred support**" OR "**user centred planning**" OR (**cash** within 2 **care**) OR (**cash** within 2 **counseling**) OR (**cash** within 2 **counselling**) OR "**individual budgets** " OR "**cash assistance**" OR "**attendance allowance**" OR "**Welfare benefit***" OR "**Welfare system***" OR "**Income support**" OR "**Benefit payment***" OR "**Social securit***" OR "**Mobility allowance***" OR "**Sickness benefit***" OR "**Invalidity benefit***" OR "**Disability benefit***" OR "**Respite care**" OR "**integrated service***" OR "**Services integration**" OR "**Social planning**" OR "**Reablement**" OR "re-ablement" OR "Re-enablement" OR "**Response Services**" OR "**Continuing Care**" OR "**transitional services**" OR "**Rehabilitation**" OR "**telerehabilitation**" OR "**electronic aids**" OR ("**electronic devices**" near **living**) OR (**aids** near "**daily living**") OR "**Assistive devices**" OR "**smart home***" OR "**Smart hous***" OR "**Smart technolog***" OR (**robot*** near **assist***) OR (**home** near **telecare**) OR (**home** near **telepresence**) OR "**befriending**" OR "**mentoring**" OR (**Control** within 2 "**daily life**") OR "**Independent living**" OR "**Living independently**" OR "**promote independence**" OR (**living** within 2 **home**) OR "**employment**" OR "**Live independently**" OR "**Independently live**" OR "**Independent lives**" OR "**Assisted living**" OR "**Assisted home***" OR "**Supported living**" OR "**social assistance**" OR "**socially assistive**" OR "**Promoting independence**" OR "**Carer consultation***" OR "**Person centred approach***" OR "**Person centred planning**")) or(**TI**= ((**Reduc*** within 2 (**admission*** or **hospitali***)) OR (**Delay*** within 2 (**admission*** or **hospitali***)) OR "**admission* avoid***" OR "**Hospital avoid***" OR "**Assisted discharge**" OR "**Reablement**" OR ("**Low-level**" within 2 (**service*** or **support** or **care** or **intervention***)) OR ("**Low level**" within 2 (**service*** or **support** or **care** or **intervention***)) OR ("**Low intensity**" within 2 (**service*** or **support** or **care** or **intervention***)) OR ("**Low-intensity**" within 2 (**service*** or **support** or **care** or **intervention***)) OR "**Intermediate care**" OR "**Preventive practice***" OR "**Maintain* independ***" OR "**Independent living**" OR "**Falls prevention**" OR "**Injur* prevention**" OR "**Accident* prevention**" OR (**Prevent*** within 1 (**fall*** or **injur*** or **accident***)) OR "**symptom relief**" OR "**symptom reduction**" OR "**Personal care**" OR "**Social participation**" OR **Accommodation** OR "**Delaying dependency**" OR "**Regaining independen***" OR ("**reducing need**" within 2 "**intensive services**") OR "**user satisfaction**" OR "**customer satisfaction**" OR "**consumer satisfaction**" OR "**client satisfaction**" OR (**experience*** within 2 **care**) OR (**experience*** within 2 **support**) OR "**user experience***" OR "**care* experience***" OR "**customer experience***" OR "**client experience***" OR "**consumer experience***" OR **dignity** OR **respect** OR "**service quality**" OR "**service provision**" OR "**quality within 2 care***" OR (**carer*** within 2 **involv***) OR "**person centred**" OR "**client centred**" OR "**user involvement**" OR **Theft*** OR **Steal** OR **stealing** OR **Fraud*** OR **Assault*** OR **Rape** OR **Neglect*** OR "Self-**neglect***" OR **Harass*** OR **violence** OR **Victim*** OR "**Ill treat***" OR "**Ill treat***" OR **Mistreat*** OR **Maltreat*** OR **Safeguard*** OR (**Prevent*** within 3 **Harm**) OR **Exploit*** OR **Crime*** OR (**Abuse*** near (**physical*** OR **emotion*** OR **sex*** OR **verbal*** OR **financ*** OR **violen***)))) or(AB= ((**Reduc*** within 2 (**admission*** or **hospitali***)) OR (**Delay*** within 2 (**admission*** or **hospitali***)) OR "**admission* avoid***" OR "**Hospital avoid***" OR "**Assisted discharge**" OR "**Reablement**" OR ("**Low-level**" within 2 (**service*** or **support** or **care** or **intervention***)) OR ("**Low level**" within 2 (**service*** or **support** or **care** or **intervention***)) OR ("**Low intensity**" within 2 (**service*** or **support** or **care** or **intervention***)) OR ("**Low-intensity**" within 2 (**service*** or **support** or **care** or **intervention***)) OR "**Intermediate care**" OR "**Preventive practice***" OR "**Maintain* independ***" OR "**Independent living**" OR "**Falls prevention**" OR "**Injur* prevention**" OR "**Accident* prevention**" OR (**Prevent*** within 1 (**fall*** or **injur*** or **accident***)) OR "**symptom relief**" OR "**symptom reduction**" OR "**Personal care**" OR "**Social participation**" OR **Accommodation** OR "**Delaying dependency**" OR "**Regaining independen***" OR ("**reducing need**" within 2 "**intensive services**") OR "**user satisfaction**" OR "**customer satisfaction**" OR "**consumer satisfaction**" OR "**client satisfaction**" OR (**experience*** within 2 **care**) OR (**experience*** within 2 **support**) OR "**user experience***" OR "**care* experience***" OR "**customer experience***" OR "**client experience***" OR "**consumer experience***" OR **dignity** OR **respect** OR "**service quality**" OR "**service provision**" OR "**quality within 2 care***" OR (**carer*** within 2 **involv***) OR "**person centred**" OR "**client centred**" OR "**user involvement**" OR **Theft*** OR **Steal** OR **stealing** OR **Fraud*** OR **Assault*** OR **Rape** OR **Neglect*** OR "Self-**neglect***" OR **Harass*** OR **violence** OR **Victim*** OR "**Ill treat***" OR "**Ill treat***" OR **Mistreat*** OR **Maltreat*** OR **Safeguard*** OR (**Prevent*** within 3 **Harm**) OR **Exploit*** OR **Crime*** OR (**Abuse*** near (**physical*** OR **emotion*** OR **sex*** OR **verbal*** OR **financ*** OR **violen***)))))

And

[Free-text search for social care populations] ((**TI**=((**Carer*** OR **Caregiv*** OR "**informal care**" OR "**informal caring**" OR "**unpaid care**" OR "**unpaid caring**" OR **caretak*** OR (**care** near **taker***) OR (**care** within 1 **taking**) OR (**families** near **support**) OR ((**parent** or **parents** or **mother** or **mothers** or **father** or **fathers**) near (**care** OR **caring** OR **support** OR **supporting**)) OR (**sons** or **daughters** or **friends**) near (**care** OR **caring** OR **support** OR **supporting**)) OR ((**husband*** or **wives** or **wife** or **spouse*** or **grandparent*** or **grandchild*** or **neighbour*** or **relatives**) near **care**))) or(AB=((**Carer*** OR **Caregiv*** OR "**informal care**" OR "**informal caring**" OR "**unpaid care**" OR "**unpaid caring**" OR **caretak*** OR (**care** near **taker***) OR (**care** within 1 **taking**) OR (**families** near **support**) OR ((**parent** or **parents** or **mother** or **mothers** or **father** or **fathers**) near (**care** OR **caring** OR **support** OR **supporting**)) OR (**sons** or **daughters** or **friends**) near (**care** OR **caring** OR **support** OR **supporting**)) OR ((**husband*** or **wives** or **wife** or **spouse*** or **grandparent*** or **grandchild*** or **neighbour*** or **relatives**) near **care**))) or(**TI**= ((**Old*** within 1 (**people*** or **patient*** or **adult*** or "**service user***" or **person*** or **men** or **women** or **male*** or **female*** or **community** or **communities** or **population*** or **age*** or **resident*** or **citizen***)) OR **seniors** OR (**senior** within 1 (**patient*** or **adult*** or "**service user***" or **person*** or **men** or **women** or **male*** or **female*** or **community** or **communities** or **population*** or **age*** or **resident*** or **citizen***)) OR **elder*** OR **geriatric*** OR **Pensioner*** OR **Frail** OR "**Nursing home resident***" OR "**late life**" OR "**later life**" OR "**late-life**" OR "**old old**" OR "**Oldest old**" OR "**pension* age**")) or(AB= ((**Old*** within 1 (**people*** or **patient*** or **adult*** or "**service user***" or **person*** or **men** or **women** or **male*** or **female*** or **community** or **communities** or **population*** or **age*** or **resident*** or **citizen***)) OR **seniors** OR (**senior** within 1 (**patient*** or **adult*** or "**service user***" or **person*** or **men** or **women** or **male*** or **female*** or **community** or **communities** or **population*** or **age*** or **resident*** or **citizen***)) OR **elder*** OR **geriatric*** OR **Pensioner*** OR **Frail** OR "**Nursing home resident***" OR "**late life**" OR "**later life**" OR "**late-life**" OR "**old old**" OR "**Oldest old**" OR "**pension* age**")) or(**TI** (**aged** within 1 (**65** or **70** or **75** or **80** or **85**))) or(**TI**= (**aged** within 1 (**65** or **70** or **75** or **80** or **85**))) or(**TI**= ("**older than 65**" OR "**older than 70**" OR "**older than 75**" OR "**older than 80**" OR "**older than 85**")) or(AB= ("**older than 65**" OR "**older than 70**" OR "**older than 75**" OR "**older than 80**" OR "**older than 85**")) or(**TI**= ("**Substance abus***" OR "**Drug user***" OR "**Drug Habituation**" OR "**Drug Use Disorder***" OR "**Substance Use Disorder***" OR "**Drug Dependenc***" OR "**Withdrawal Syndrome***" OR "**Dependency disorder***" OR ((**drug** or **substance**) within 1 (**abuse*** or **misuse** or **depend*** or **addict***)) OR **Schizo*** OR **Catatonia** OR **catatonic** OR **Depression** OR "Bi-polar" OR **bipolar** OR **Mania** OR **Hypomania** OR **Cyclothymia** OR **Dysthymia** OR "**Mood disorder***" OR "**Depressive Disorder***" OR **OCD** OR "**obsessive compulsive**" OR "**Eating Disorder***" OR **bulimi*** OR "**Bulimia Nervosa**" OR **anorexi*** OR "**anorexia nervosa**" OR "Binge-**Eating Disorder***" OR "**Personality disorder***" OR "**Affective Disorder***" OR "**Neurotic Disorder***" OR "**Antisocial Personality Disorder***" OR "**Borderline Personality Disorder***" OR "**Compulsive Personality Disorder***" OR "**Dependent Personality Disorder***" OR "**Histrionic Personality Disorder***" OR "**Paranoid Personality Disorder***" OR "Passive-Aggressive **Personality Disorder***" OR "**Schizoid Personality Disorder***" OR "**Schizotypal Personality Disorder***" OR (**anankastic** within 1 **person**) OR (**Asocial** within 1 **person**) OR (**Antisocial** within 1 **person**) OR (**Avoidant** within 1 **person**) OR (**Borderline** within 1 **person**) OR (**Dependent** within 1 **person**) OR (**Dissocial** within 1 **person**) OR (**Histrionic** within 1 **person**) OR (**Narcissistic** within 1 **person**) OR (**Obsessive** within 1 **person**) OR (**Compulsive** within 1 **person**) OR (**Paranoid** within 1 **person**) OR ("Passive-aggressive" within 1 **person**) OR (**Sadomasochistic** within 1 **person**) OR (**Disorders N1** ("**Psychotic Feature***")) OR "**Capgras Syndrome**" OR "**Paranoid Disorder***" OR "**Psychotic Disorder***" OR ((**Sexual** OR **Gender**) within 1 **Disorder***) OR (**Disorder*** within 1 "**Sex Development**") OR ("**Sexual Dysfunction***" **N1 Psychological**) OR "**Somatoform Disorder***" OR "**Body Dysmorphic Disorder***" OR "**Conversion Disorder***" OR "**Hypochondriasis**" OR "**Neurasthenia**" OR "**Adjustment Disorder***" OR "**Anxiety Disorder***" OR "**Impulse Control Disorder***" OR "**Reactive Attachment Disorder***" OR "**Dissociative Disorder***" OR "**Multiple Personality Disorder***" OR "**Cognitive Disorder***" OR "**Stress Disorder***" OR "**Cognition Disorder***" OR "**Consciousness Disorder***" OR "**Panic Disorder***" OR "**Phobic Disorder***" OR "**adjustment disorder***" OR "**overactive disorder***" OR "**disintegrative disorder***" OR "**pervasive developmental disorder***" OR "**hyperkinetic disorder***" OR **Dementia** OR **Alzheimer*** OR **amnesi*** OR **delirium** OR **hallucinosis** OR **delusional** OR **asthenic** OR "**emotionally labile**" OR **Posttraumatic** OR "**post traumatic**" OR **postencephalitic** OR **postconcussion*** OR "**trance disorder***" OR "**possession disorder***" OR (**anxious** within 1 (**problem*** OR **difficult*** or **disorder*** or **ill***)) OR (**anxiety** within 1 (**problem*** OR **difficult*** or **disorder*** or **ill***)) OR "**multiple personalit***" OR **dissociate** OR **neurasthenia** OR **depersonali?ation** OR **derealisation** OR **derealization** OR **suicid*** OR **parasuicid*** OR "**Self harm**" OR "**self injur***" OR **Coprophagia** OR "**Female Athlete Triad Syndrome**" OR "**Pica**" OR "**Factitious Disorder***" OR "**Munchausen Syndrome**" OR "**Trichotillomania**" OR "**Agoraphobia**" OR "**Neurocirculatory Asthenia**" OR **hebephreni*** OR **oligophreni*** OR **somatisation** OR (**psychiatric** within 1 (**problem*** OR **difficult*** or **disorder*** or **illness**)) OR **Psychosis** OR ("**mental health**" within 1 (**problem*** OR **difficult*** or **disorder*** or **ill***)) OR "**psychological disturbance***" or "**psychologically disturbed**" OR **neuros*** OR "**psychological stress**" OR "**psychological distress**" OR "**mental health status**" OR "**mental stress**" OR "**mental health patients**" OR "**mental health patient**" OR "**mental health treatment**" OR "**mentally ill**" OR "**severe stress**" OR **comorbid***)) or(AB= ("**Substance abus***" OR "**Drug user***" OR "**Drug Habituation**" OR "**Drug Use Disorder***" OR "**Substance Use Disorder***" OR "**Drug Dependenc***" OR "**Withdrawal Syndrome***" OR "**Dependency disorder***" OR ((**drug** or **substance**) within 1 (**abuse*** or **misuse** or **depend*** or **addict***)) OR **Schizo*** OR **Catatonia** OR **catatonic** OR **Depression** OR "Bi-polar" OR **bipolar** OR **Mania** OR **Hypomania** OR **Cyclothymia** OR **Dysthymia** OR "**Mood disorder***" OR "**Depressive Disorder***" OR **OCD** OR "**obsessive compulsive**" OR "**Eating Disorder***" OR **bulimi*** OR "**Bulimia Nervosa**" OR **anorexi*** OR "**anorexia nervosa**" OR "Binge-**Eating Disorder***" OR "**Personality disorder***" OR "**Affective Disorder***" OR "**Neurotic Disorder***" OR "**Antisocial Personality Disorder***" OR "**Borderline Personality Disorder***" OR "**Compulsive Personality Disorder***" OR "**Dependent Personality Disorder***" OR "**Histrionic Personality Disorder***" OR "**Paranoid Personality Disorder***" OR "Passive-Aggressive **Personality Disorder***" OR "**Schizoid Personality Disorder***" OR "**Schizotypal Personality Disorder***" OR (**anankastic** within 1 **person**) OR (**Asocial** within 1 **person**) OR (**Antisocial** within 1 **person**) OR (**Avoidant** within 1 **person**) OR (**Borderline** within 1 **person**) OR (**Dependent** within 1 **person**) OR (**Dissocial** within 1 **person**) OR (**Histrionic** within 1 **person**) OR (**Narcissistic** within 1 **person**) OR (**Obsessive** within 1 **person**) OR (**Compulsive** within 1 **person**) OR (**Paranoid** within 1 **person**) OR ("Passive-aggressive" within 1 **person**) OR (**Sadomasochistic** within 1 **person**) OR (**Disorders N1** ("**Psychotic Feature***")) OR "**Capgras Syndrome**" OR "**Paranoid Disorder***" OR "**Psychotic Disorder***" OR ((**Sexual** OR **Gender**) within 1 **Disorder***) OR (**Disorder*** within 1 "**Sex Development**") OR ("**Sexual Dysfunction***" **N1 Psychological**) OR "**Somatoform Disorder***" OR "**Body Dysmorphic Disorder***" OR "**Conversion Disorder***" OR "**Hypochondriasis**" OR "**Neurasthenia**" OR "**Adjustment Disorder***" OR "**Anxiety Disorder***" OR "**Impulse Control Disorder***" OR "**Reactive Attachment Disorder***" OR "**Dissociative Disorder***" OR "**Multiple Personality Disorder***" OR "**Cognitive Disorder***" OR "**Stress Disorder***" OR "**Cognition Disorder***" OR "**Consciousness Disorder***" OR "**Panic Disorder***" OR "**Phobic Disorder***" OR "**adjustment disorder***" OR "**overactive disorder***" OR "**disintegrative disorder***" OR "**pervasive developmental disorder***" OR "**hyperkinetic disorder***" OR **Dementia** OR **Alzheimer*** OR **amnesi*** OR **delirium** OR **hallucinosis** OR **delusional** OR **asthenic** OR "**emotionally labile**" OR **Posttraumatic** OR "**post traumatic**" OR **postencephalitic** OR **postconcussion*** OR "**trance disorder***" OR "**possession disorder***" OR (**anxious** within 1 (**problem*** OR **difficult*** or **disorder*** or **ill***)) OR (**anxiety** within 1 (**problem*** OR **difficult*** or **disorder*** or **ill***)) OR "**multiple personalit***" OR **dissociate** OR **neurasthenia** OR **depersonali?ation** OR **derealisation** OR **derealization** OR **suicid*** OR **parasuicid*** OR "**Self harm**" OR "**self injur***" OR **Coprophagia** OR "**Female Athlete Triad Syndrome**" OR "**Pica**" OR "**Factitious Disorder***" OR "**Munchausen Syndrome**" OR "**Trichotillomania**" OR "**Agoraphobia**" OR "**Neurocirculatory Asthenia**" OR **hebephreni*** OR **oligophreni*** OR **somatisation** OR (**psychiatric** within 1 (**problem*** OR **difficult*** or **disorder*** or **illness**)) OR **Psychosis** OR ("**mental health**" within 1 (**problem*** OR **difficult*** or **disorder*** or **ill***)) OR "**psychological disturbance***" or "**psychologically disturbed**" OR **neuros*** OR "**psychological stress**" OR "**psychological distress**" OR "**mental health status**" OR "**mental stress**" OR "**mental health patients**" OR "**mental health patient**" OR "**mental health treatment**" OR "**mentally ill**" OR "**severe stress**" OR **comorbid***)) or(**TI**= (**disabled** OR **disabilit*** OR **handicap*** or "**physical* impair***" OR "**functional* impair***" OR **incapacitated** OR "**physically challenged**" OR "**wheelchair user***" or "**sensory impairment***" OR "**hearing impair***" OR "**auditory impair***" OR "**Speech Impair***" OR "**speech impediment***" OR "**visual impairment***" OR "**visually impaired**" OR "**hearing loss**" OR **deaf*** OR **blindness** OR "**Vision Disorder***" OR "**Partial* sight***" OR **Cataract*** OR "**Macular degeneration**" OR **mutism** OR **mute** OR (**Chronic** within 1 (**condition*** or **illness*** or **disease***)) OR ("long-term" within 1 (**condition*** or **illness*** or **disease*** or **sick***)) OR **Stroke** OR **Cancer** OR **HIV** OR "**Mobility impair***" OR "**Impaired mobility**" OR **Arthritis** OR **osteoarthritis** OR "**Cerebal Palsy**" OR "**Cystic Fybrosis**" OR **Polio** OR "**Spina Bifida**" OR "**Spinal Injury**" OR "**Paraplegic***" OR **Quadriplegic*** OR **Tetraplegic*** OR "**Muscular Dystrophy**" OR **Parkinson*** OR **Huntington*** OR **Lupus** OR "**Motor Neurone**" OR "**Multiple Sclerosis**" OR "**Post-injury**" OR "**post injury**" OR "**Head Injur***" OR "**brain injur***" OR "**Limbless**" OR **Amputee*** OR "**spinal cord injur***" OR "**Back pain**")) or(AB= (**disabled** OR **disabilit*** OR **handicap*** or "**physical* impair***" OR "**functional* impair***" OR **incapacitated** OR "**physically challenged**" OR "**wheelchair user***" or "**sensory impairment***" OR "**hearing impair***" OR "**auditory impair***" OR "**Speech Impair***" OR "**speech impediment***" OR "**visual impairment***" OR "**visually impaired**" OR "**hearing loss**" OR **deaf*** OR **blindness** OR "**Vision Disorder***" OR "**Partial* sight***" OR **Cataract*** OR "**Macular degeneration**" OR **mutism** OR **mute** OR (**Chronic** within 1 (**condition*** or **illness*** or **disease***)) OR ("long-term" within 1 (**condition*** or **illness*** or **disease*** or **sick***)) OR **Stroke** OR **Cancer** OR **HIV** OR "**Mobility impair***" OR "**Impaired mobility**" OR **Arthritis** OR **osteoarthritis** OR "**Cerebal Palsy**" OR "**Cystic Fybrosis**" OR **Polio** OR "**Spina Bifida**" OR "**Spinal Injury**" OR "**Paraplegic***" OR **Quadriplegic*** OR **Tetraplegic*** OR "**Muscular Dystrophy**" OR **Parkinson*** OR **Huntington*** OR **Lupus** OR "**Motor Neurone**" OR "**Multiple Sclerosis**" OR "**Post-injury**" OR "**post injury**" OR "**Head Injur***" OR "**brain injur***" OR "**Limbless**" OR **Amputee*** OR "**spinal cord injur***" OR "**Back pain**")) or(**TI**= ((**learning** near (**difficult*** or **disable*** or **disabilit*** or **disorder*** or **deficien*** or **incapacity** or **handicap*** or **impair*** or **retard***)) OR (**mental*** near (**difficult*** or **disable*** or **disabilit*** or **disorder*** or **deficien*** or **incapacity** or **handicap*** or **impair*** or **retard*** or **intellect***)) OR (**intellect*** near (**difficult*** or **disable*** or **disabilit*** or **disorder*** or **deficien*** or **incapacity** or **handicap*** or **impair*** or **retard***)) OR (**cognitive*** near (**difficult*** or **disable*** or **disabilit*** or **disorder*** or **deficien*** or **incapacity** or **handicap*** or **impair*** or **retard***)) OR (**developmental*** near (**delay*** OR **difficult*** or **disable*** or **disabilit*** or **disorder*** or **deficien*** or **incapacity** or **handicap*** or **impair*** or **retard***)) OR "**subnormal intell***" OR "**down* syndrome**" OR **Autis*** OR "**Rett?s syndrome**" OR (**learn*** near **problem***) OR (**behav*** near **problem***) OR "**behav* disorder**" OR "**adhd**" OR "**asperger***" OR "**fragile within 1 syndrome**" OR (**attention*** within 1 **deficit***) OR **hyperactiv*** OR "**conduct disorder***" OR (**conduct** near **problem***))) or(AB= ((**learning** near (**difficult*** or **disable*** or **disabilit*** or **disorder*** or **deficien*** or **incapacity** or **handicap*** or **impair*** or **retard***)) OR (**mental*** near (**difficult*** or **disable*** or **disabilit*** or **disorder*** or **deficien*** or **incapacity** or **handicap*** or **impair*** or **retard*** or **intellect***)) OR (**intellect*** near (**difficult*** or **disable*** or **disabilit*** or **disorder*** or **deficien*** or **incapacity** or **handicap*** or **impair*** or **retard***)) OR (**cognitive*** near (**difficult*** or **disable*** or **disabilit*** or **disorder*** or **deficien*** or **incapacity** or **handicap*** or **impair*** or **retard***)) OR (**developmental*** near (**delay*** OR **difficult*** or **disable*** or **disabilit*** or **disorder*** or **deficien*** or **incapacity** or **handicap*** or **impair*** or **retard***)) OR "**subnormal intell***" OR "**down* syndrome**" OR **Autis*** OR "**Rett?s syndrome**" OR (**learn*** near **problem***) OR (**behav*** near **problem***) OR "**behav* disorder**" OR "**adhd**" OR "**asperger***" OR "**fragile within 1 syndrome**" OR (**attention*** within 1 **deficit***) OR **hyperactiv*** OR "**conduct disorder***" OR (**conduct** near **problem***))))

And

[Free-text search for systematic reviews] (**TI**=(**systematic** within 2 **review**) OR **TI**=(**systematic** within 2 **review***) OR **TI**="meta-analysis" OR AB= (**systematic** within 2 **review**) OR AB= (**systematic** within 2 **review***) OR AB="meta-analysis" OR DE="**Review articles**")

## Data extraction tool for meta-review synthesis

| **Data extraction questions**  **and guidance** | **Answers, further**  **sub-questions and guidance** |
| --- | --- |
| 1. **What type of studies are included and are they quality appraised?** *This question is to get at the quality of the reviews and the quality of the studies that they contain* | - - **RCTs only - with QA**   - **RCTs only - no QA**   - **CTs and RCTs - no QA**   - **CTs and RCTs - with QA** |
| 1. **Review question/Aims** (*Please copy/write in authors description of the review question/aims)* | - - Details |
| **3**. **Are the populations / interventions / outcomes covered in the SR all ASCOF relevant?** (*This question is to capture whether the reviews contain evidence that is NOT relevant to our review question.)* | - - **Yes - the whole review is relevant** (*use this code if all the evidence reported in the review is relevant to ASCO)*   - **No - some of the reviews evidence is not relevant** (provide details)   - non-Social care interventions included (details)  - non-ASCOF outcomes measured (details)  - non-Social care populations included (details) |
| **4.** **Which outcomes are usable summary statements available for?** (*Please check the boxes - and write in the details of the relevant outcomes under each category)* | - - **Quality of life:**      - Generic: (*capture HRQL - and other generic or overall measures of QoL*     - Activity/Mobility (c*apture measures of ADL)*     - Engagement (*capture outcomes relating to social engagement/interaction/employment etc)*     - Material (*capture outcomes about - housing, food etc)*     - Dignity/control/respect   - **Prevention** (e.g. *delaying dependency, regaining independence, reducing need for intensive services)*      - Prevention - illness/events     - Prevention - service use   - **Satisfaction** (e.g. *service user satisfaction*, *experiences of information/advice*, *perception of whether service respects dignity and/or is tailored to individual needs/preferences)*      - Details   - **Safeguarding** (e.g. *protecting service users from ill treatment, including the impairment of health or physical, intellectual, emotional, social or behavioural development and other forms of neglect and unlawful conducting*      - Details   - **Cost** (any economic / cost data) |
| **5.** **Which interventions are usable summary statements available for?** *Please provide further details in text box* | - - **Alternative therapies**   - **Assistive devices**   - **Physical activity**   - **Occupational therapy**   - **Needs assessment/case management**   - **Peer support/peer education**   - **Personal assistance**   - **Supported employment**   - **Supported housing**   - **Other** |
| **6. What do comparison/control groups receive?** *Please provide details of what the control / comparison groups receive – using the authors description – e.g. usual care, waitlist control.* | - - **Details** |
| 1. **How many relevant summary statements does this review contain** | - - **Details** |
| 1. **Prevention Summary statements (details)** *Please copy/write in the details of usable summary statements relating to prevention* | - - **N/A no prevention summary statements (SS)**      - - **Prevention -** What do authors conclude about impact on prevention?     - Evidence of positive impact     - No evidence of difference     - Evidence of negative impact / harm     - Evidence inconclusive     - Evidence insufficient   - **How many studies are the prevention SSs based on?** *(Please details of numbers for each prevention summary statement)*     - 0-5     - 6-10     - 11+ - **Quality of prevention evidence** *(what do authors conclude about trustworthiness of evidence for this summary statement?)*    - - N/A - no assessment of quality     - Prevention data = High quality evidence   (*Use this code if the authors a) judge that all studies included in the review are of high quality b) refer to the particular studies underpinning SS1 as being high quality)*   - - - Prevention data = Concerns about quality of evidence   (*Use this code if a) authors have concerns about the quality of all evidence included in the review b) have concerns about the particular studies contributing to this particular summary statement*   - **Do the reviewers agree with prevention conclusions?** (*Use this code to voice any concerns you have)*    - - Yes (*Use this code if you agree that authors conclusions are valid*     - No (*Use this code if you have concerns about the validity of SS’s - and explain your concerns)* |
| **8. QoL Summary statements (details)** *Please copy/write in the details of usable summary statements about QoL* | - - **N/A no QoL summary statements (SS)**      - - **QoL -** What do authors conclude about impact on prevention?     - Evidence of positive impact     - No evidence of difference     - Evidence of negative impact / harm     - Evidence inconclusive     - Evidence insufficient   - **How many studies are the QoL SSs based on?** *(Please details of numbers for each prevention summary statement)*     - 0-5     - 6-10     - 11+ - **Quality of QoL evidence** *(what do authors conclude about trustworthiness of evidence for this summary statement?)*    - - N/A - no assessment of quality     - QoL data = High quality evidence   (*Use this code if the authors a) judge that all studies included in the review are of high quality b) refer to the particular studies underpinning SS1 as being high quality)*   - - - QoL data = Concerns about quality of evidence   (*Use this code if a) authors have concerns about the quality of all evidence included in the review b) have concerns about the particular studies contributing to this particular summary statement*   - **Do the reviewers agree with QoL conclusions?** (*Use this code to voice any concerns you have)*    - - Yes (*Use this code if you agree that authors conclusions are valid*     - No (*Use this code if you have concerns about the validity of SS’s - and explain your concerns* |
| **9. Satisfaction summary statements (details)** *Please copy/write in the details of usable summary statements about* **satisfaction** | - - **N/A no satisfaction summary statements (SS)**      - - **Satisfaction -** What do authors conclude about impact on prevention?     - Evidence of positive impact     - No evidence of difference     - Evidence of negative impact / harm     - Evidence inconclusive     - Evidence insufficient   - **How many studies are the satisfaction SSs based on?** *(Please details of numbers for each prevention summary statement)*     - 0-5     - 6-10     - 11+ - **Quality of satisfaction evidence** *(what do authors conclude about trustworthiness of evidence for this summary statement?)*    - - N/A - no assessment of quality     - Satisfaction data = High quality evidence   (*Use this code if the authors a) judge that all studies included in the review are of high quality b) refer to the particular studies underpinning SS1 as being high quality)*   - - - Satisfaction data = Concerns about quality of evidence   (*Use this code if a) authors have concerns about the quality of all evidence included in the review b) have concerns about the particular studies contributing to this particular summary statement*   - **Do the reviewers agree with satisfaction conclusions?** (*Use this code to voice any concerns you have)*    - - Yes (*Use this code if you agree that authors conclusions are valid*   No (*Use this code if you have concerns about the validity of SS’s - and explain your concerns* |
| **10. Safeguarding summary statements** | - - **N/A no safeguarding summary statements (SS)**      - - **Safeguarding -** What do authors conclude about impact on prevention?     - Details   - **How many studies are the safeguarding SSs based on?** *(Please details of numbers for each prevention summary statement)*     - 0-5     - 6-10     - 11+ - **Quality of Safeguarding evidence** *(what do authors conclude about trustworthiness of evidence for this summary statement?)*    - - N/A - no assessment of quality     - Safeguarding data = High quality evidence   (*Use this code if the authors a) judge that all studies included in the review are of high quality b) refer to the particular studies underpinning SS1 as being high quality)*   - - - Safeguarding data = Concerns about quality of evidence   (*Use this code if a) authors have concerns about the quality of all evidence included in the review b) have concerns about the particular studies contributing to this particular summary statement*   - **Do the reviewers agree with Safeguarding conclusions?** (*Use this code to voice any concerns you have)*    - - Yes (*Use this code if you agree that authors conclusions are valid*     - No (*Use this code if you have concerns about the validity of SS’s - and explain your concerns* |

# Design and quality of systematic reviews

## Quality of life outcomes

| **Review** | **Quality of life outcomes (Qol)** | **Included studies and summary statements (SS)** | **Review methods** | **Conclusions** |
| --- | --- | --- | --- | --- |
| Allison (2011) | QoL  ADL: physical functioning | **Design and quality**: RCTs only – with QA  **Author conclusions about trustworthiness of evidence**: Concerns about quality of evidence  **How many studies are quality of life SS based on?**  QoL: 4; ADL: 4 | Narrative synthesis | **Author conclusions about impact on QoL**:  No evidence of difference  **Do the reviewers agree with QoL e conclusions?:**  Yes |
| Arbesman (2011) | Engagement | **Design and quality**: CTs and RCTs – with QA  **Author conclusions about trustworthiness of evidence**  Concerns about quality of evidence  **How many studies are quality of life SS based on?**  Supported education: 2; SE plus cognitive skills training: 1; Programmes related to homemaking: 1; Social and daily living skills: 3 + 3 | Narrative synthesis | **Author conclusions about impact on QoL**:  Evidence of positive impact  **Do the reviewers agree with QoL conclusions?**  Yes: Improvements shown for 1) Supported education and 2) Social and daily living skills interventions  No: homemaking: evidence inconclusive (1 study) |
| Baillet (2010) | QoL | **Design and quality**: RCTs only – with QA  **Author conclusions about trustworthiness of evidence**  High-quality evidence  **How many studies are quality of life SS based on?** 5 | Meta-analysis | **Author conclusions about impact on QoL**:  Evidence of positive impact  **Do the reviewers agree with QoL conclusions?**  Yes |
| Bartlo (2011) | QoL | **Design and quality**: RCTs only – with QA  **Author conclusions about trustworthiness of evidence**  High-quality evidence  **How many studies are quality of life SS based on?** 4 RCTs | Narrative synthesis | **Author conclusions about impact on QoL**:  Evidence of positive impact  **Do the reviewers agree with Qol conclusions?**  Yes |
| Bender (2011) | ADL | **Design and quality**: RCTs only – with QA  **Author conclusions about trustworthiness of evidence**  Concerns about quality of evidence  **How many studies are quality of life SS based on?** 1 | Narrative synthesis | **Author conclusions about impact on QoL**: Evidence of positive impact: Internet-based peer support; No evidence of difference: Peer support with complementary alternative medicine  **Do the reviewers agree with QoL conclusions?**  No: The evidence is inconclusive and low quality |
| Bond (2008) | Engagement: employment outcomes | **Design and quality**: RCTs only – no QA  **Author conclusions about trustworthiness of evidence**  N/A – no assessment of quality  **How many studies are quality of life SS based on?**  11 | Narrative synthesis | **Author conclusions about impact on QoL**: Evidence of positive impact: employment  No evidence of difference: job tenure and other employment outcomes  **Do the reviewers agree with QoL conclusions?**  Yes |
| Bradt (2011) | QoL | **Design and quality**: RCTs only – with QA  **Author conclusions about trustworthiness of evidence**  High-quality evidence  **How many studies are quality of life SS based on?** 1 | Narrative synthesis | **Author conclusions about impact on QoL**:  Evidence of positive impact  **Do the reviewers agree with QoL conclusions?**  No: inconclusive evidence (only 1 one RCT) |
| Chatterton (2010) | Engagement: social functioning | **Design and quality**: CTs and RCTs – no QA  **Author conclusions about trustworthiness of evidence**  N/A – no assessment of quality  **How many studies are quality of life Summary Statements based on?** 2 | Narrative synthesis | **Author conclusions about impact on QoL** Evidence of positive impact  **Do the reviewers agree with QoL conclusions?**  No: Evidence is inconclusive. Only 2 studies provide that and have not been quality appraised |
| Daniels (2008) | ADL | **Design and quality**: RCTs only – with QA  **Author conclusions about trustworthiness of evidence**  High-quality evidence  **How many studies are quality of life Summary Statements based on?** 9 | Narrative synthesis | **Author conclusions about impact on QoL** Evidence inconclusive  **Do the reviewers agree with QoL conclusions?**  Yes: there are mixed findings – agree with the 'no overall evidence' |
| Dickson (2008) | QoL  ADL  Engagement | **Design and quality**: CTs and RCTs – with QA  **Author conclusions about trustworthiness of evidence**  High-quality evidence  **How many studies are quality of life SS based on?** 7 | Narrative synthesis | **Author conclusions about impact on QoL**  No evidence of difference; Evidence inconclusive **Do the reviewers agree with QoL conclusions?**  Yes |
| Dixon (2007) | QoL  ADL | **Design and quality**: RCTs only – with QA  **Author conclusions about trustworthiness of evidence**  Poor-quality evidence  **How many studies are quality of life SS based on?** 2 | Narrative synthesis | **Author conclusions about impact on QoL** No Evidence inconclusive  **Do the reviewers agree with QoL conclusions?**  Yes |
| Floyd (2010) | QoL | **Design and quality**: CTs and RCTs – with QA  **Author conclusions about trustworthiness of evidence**  Concerns about quality of evidence  **How many studies are quality of life Summary Statements based on?** 12 | Meta-analysis | **Author conclusions about impact on QoL** Evidence of positive impact QoL; No evidence of difference: that group exercise is more effective than individual programmes  **Do the reviewers agree with QoL conclusions?**  Yes |
| Forbes (2008) | ADL | **Design and quality**: RCTs only – with QA  **Author conclusions about trustworthiness of evidence**  Concerns about quality of evidence  **How many studies are quality of life SS based on?** 2 | Meta-analysis | **Author conclusions about impact on QoL** Evidence inconclusive  **Do the reviewers agree with QoL conclusions?**  Yes insufficient evidence |
| Forster (2009) | ADL | **Design and quality**: RCTs only – with QA  **Author conclusions about trustworthiness of evidence**  Concerns about quality of evidence  **How many studies are quality of life SS based on?** 36 | Narrative synthesis | **Author conclusions about impact on QoL** Evidence of positive impact  **Do the reviewers agree with QoL conclusions?**  Yes |
| Foster (2007) | QoL | **Design and quality**: RCTs only – with QA  **Author conclusions about trustworthiness of evidence**  High-quality evidence  **How many studies are quality of life SS based on?** 3 | Meta-analysis | **Author conclusions about impact on QoL**:  No evidence of difference  **Do the reviewers agree with QoL conclusions?**  Yes |
| Gillison (2009) | QoL  ADL | **Design and quality**: RCTs only – no QA  **Author conclusions about trustworthiness of evidence**  N/A – no assessment of quality  **How many studies are quality of life SS based on?** 47 | Meta-analysis | **Author conclusions about impact on QoL**:  Evidence of positive impact for rehabilitating from LTC; Evidence of harm for managing LTC  **Do the reviewers agree with QoL conclusions?**  Yes |
| Hall (2009) | QoL  ADL: disability and physical functioning | **Design and quality**: RCTs only – with QA  **Author conclusions about trustworthiness of evidence**  Concerns about quality of evidence  **How many studies are quality of life SS based on?**  QoL: 3; Activity/mobility: 4 for self-reported disability, 2 for physical functioning | Narrative synthesis: QoL | **Author conclusions about impact on QoL**:  Evidence of positive impact: disability: Evidence inconclusive: non-significant findings for QoL and physical functioning  **Do the reviewers agree with QoL conclusions?**  Yes: Agree with the authors evidence is ‘unclear’ |
| Hand (2011) | ADL  Physical function | **Design and quality**: RCTs only – with QA  **Author conclusions about trustworthiness of evidence**  Concerns about quality of evidence  **How many studies are quality of life SS based on?**  3 ADL: 9 physical function | Narrative synthesis | **Author conclusions about impact on QoL**:  Evidence of positive impact: ADL; No evidence of difference: physical function  **Do the reviewers agree with QoL conclusions?**  No: Reviewers concerned that no statistical information is provided to support the findings. |
| Harling (2008) | Dignity/ control/ respect Fear of falling | **Design and quality**: RCTs only – with QA  **Author conclusions about trustworthiness of evidence**  High-quality evidence  **How many studies are quality of life SSbased on?** 5 | Narrative synthesis | **Author conclusions about impact on QoL**:  Evidence of positive impact  **Do the reviewers agree with quality of life conclusions?**  Yes |
| Hauser (2010) | QoL | **Design and quality**: RCTs only – with QA  **Author conclusions about trustworthiness of evidence**  Concerns about quality of evidence  **How many studies are quality of life SS based on?** 25 | Meta-analysis | **Author conclusions about impact on QoL**:  Evidence of positive impact  **Do the reviewers agree with QoL conclusions?**  Yes |
| Jain (2010) | QoL | **Design and quality**: CTs and RCTs – with QA  **Author conclusions about trustworthiness of evidence**  High-quality evidence  **How many studies are quality of life SS based on?**  Pain: 3; Cancer: 3 | Narrative synthesis | **Author conclusions about impact on QoL**:  Evidence inconclusive  **Do the reviewers agree with QoL conclusions?**  Yes: |
| Kong Jae (2010) | QoL  ADL | **Design and quality**: RCTs only – with QA  **Author conclusions about trustworthiness of evidence**  Concerns about quality of evidence  **How many studies are quality of life SS based on?** 5 | Meta-analysis | **Author conclusions about impact on QoL**:  No evidence of difference  **Do the reviewers agree with QoL conclusions?**  Yes and no |
| Lee (2007) | QoL  ADL  Functional index | **Design and quality**: CTs and RCTs – with QA  **Author conclusions about trustworthiness of evidence**  Concerns about quality of evidence  **How many studies are quality of life SS based on?** 1 | Narrative synthesis | **Author conclusions about impact on QoL**:  Evidence of positive impact: QoL; Evidence inconclusive: overall  **Do the reviewers agree with QoL conclusions?**  Yes: Inconclusive for QoL. Only 1 low quality study |
| Legg (2007) | ADL | **Design and quality**: RCTs only – with QA  **Author conclusions about trustworthiness of evidence**  High-quality evidence  **How many studies are quality of life SS based on?** 8 | Meta-analysis | **Author conclusions about impact on QoL**:  Evidence of positive impact: activity/ mobility  **Do the reviewers agree with QoL conclusions?**  Yes |
| Lin (2011) | QoL | **Design and quality**: RCTs only – with QA  **Author conclusions about trustworthiness of evidence**  Concerns about quality of evidence  **How many studies are quality of life SS based on?** 3 | Meta-analysis | **Author conclusions about impact on QoL**:  Evidence inconclusive  **Do the reviewers agree with QoL conclusions?**  Yes |
| Lowe (2009) | QoL  ADL | **Design and quality**: RCTs only – with QA  **Author conclusions about trustworthiness of evidence**  Concerns about quality of evidence  **How many studies are quality of life SS based on?** 1 | Narrative synthesis | **Author conclusions about impact on QoL**:  Evidence of positive impact: QoL; No evidence of difference: activity/mobility: Evidence inconclusive: overall  **Do the reviewers agree with QoL conclusions?**  Yes: We would argue the evidence is inconclusive. insufficient evidence with only 1 low quality RCT, |
| Mayo-Wilson (2008) | QoL  ADL Engagement | **Design and quality**: CTs and RCTs – with QA  **Author conclusions about trustworthiness of evidence**  Concerns about quality of evidence  **How many studies are quality of life SS based on?** 2 | Narrative synthesis | **Author conclusions about impact on QoL**:  Evidence inconclusive  **Do the reviewers agree with QoL conclusions?**  Yes |
| Mont-gomery (2008) | QoL  ADL  Engagement | **Design and quality**: CTs and RCTs – with QA  **Author conclusions about trustworthiness of evidence**  High-quality evidence  **How many studies are quality of life SS based on?** 4 | Narrative synthesis | **Author conclusions about impact on QoL**:  Evidence of positive impact: activity/mobility  No evidence of difference: engagement  **Do the reviewers agree with QoL conclusions?**  Yes: Agree with authors’ cautious conclusion that the intervention 'may' help |
| Olazaran (2010) | QoL  ADL | **Design and quality**: RCTs only – with QA  **Author conclusions about trustworthiness of evidence**  Concerns about quality of evidence  **How many studies are quality of life SS based on?**  QoL: 2; Activity/mobility: 7 | Meta-analysis | **Author conclusions about impact on QoL**:  Evidence of positive impact: activity/mobility  Evidence inconclusive: QoL  **Do the reviewers agree with QoL conclusions?**  Yes |
| Padilla (2011) | QoL  ADL | **Design and quality**: CTs and RCTs – with QA  **Author conclusions about trustworthiness of evidence**  Concerns about quality of evidence  **How many studies are quality of life SS based on?**  Assistive devices: 4: OT: 4 | Narrative synthesis | **Author conclusions about impact on QoL**:  Evidence of positive impact: consistent positive effects  **Do the reviewers agree with QoL conclusions?**  Yes |
| Schuch (2011) | QoL  ADL: physical function  Engagement: social function | **Design and quality**: RCTs only – with QA  **Author conclusions about trustworthiness of evidence**  Concerns about quality of evidence  **How many studies are quality of life SS based on?** 4 | Narrative synthesis | **Author conclusions about impact on QoL**:  Evidence inconclusive:  **Do the reviewers agree with QoL conclusions?**  Yes |
| Tungpunkom (2012) | QoL  ADL  Engagement | **Design and quality**: CTs and RCTs – with QA  **Author conclusions about trustworthiness of evidence**  Concerns about quality of evidence  **How many studies are quality of life SS based on?**  Activity/mobility: 1; QoL: 1; Engagement: 1 | Narrative synthesis | **Author conclusions about impact on QoL**:  No evidence of difference  **Do the reviewers agree with QoL conclusions?**  Yes |
| Tuntland (2009) | ADL | **Design and quality**: RCTs only – with QA  **Author conclusions about trustworthiness of evidence**  Concerns about quality of evidence  **How many studies are quality of life SS based on?** 1 | Narrative synthesis | **Author conclusions about impact on QoL**:  Evidence inconclusive  **Do the reviewers agree with QoL conclusions?**  Yes |
| Vasse (2010) | Engagement | **Design and quality**:: CTs and RCTs – with QA  **Author conclusions about trustworthiness of evidence**  Concerns about quality of evidence  **How many studies are quality of life SS based on?** 10 | Meta-analysis and narrative synthesis | **Author conclusions about impact on QoL**:  Evidence of positive impact  No evidence of difference  **Do the reviewers agree with QoL conclusions?**  No: The majority of studies (8/10) show no effect. The meta-analysis of the 5 higher-quality studies shows no evidence of effect. The authors, however, seem to focus on 2 studies that did demonstrate positive effect (single task interventions) |

## Prevention outcomes

| **Review** | **Prevention outcomes** | **Included studies** | **Review methods** | **Conclusions** |
| --- | --- | --- | --- | --- |
| Allison (2011) | **Illness/ events:** Mood | **Design and quality**: RCTs only – with QA  **Author conclusions about trustworthiness of evidence**: Concerns about quality of evidence  **How many studies are prevention SS based on?**  6 | Narrative synthesis | **Author conclusions about impact on prevention**  Evidence inconclusive  **Do the reviewers agree with prevention conclusions?**  Yes |
| Bender (2011) | **Illness/events:**  Health distress | **Design and quality:** RCTs only – with QA  **Author conclusions about trustworthiness of evidence**  Concerns about quality of evidence  **How many studies are prevention SS based on?**  2 | Narrative synthesis | **Author conclusions about impact on prevention**  Evidence of positive impact  **Do the reviewers agree with prevention conclusions?**  Yes: Agree with the authors that there is 'limited but promising' evidence |
| Bradt (2011) | **Illness/events:**  Mood, distress and mental health | **Design and quality:** RCTs only – with QA  **Author conclusions about trustworthiness of evidence**  Concerns about quality of evidence  **How many studies are prevention Summary Statements based on?** 2 | Narrative synthesis | **Author conclusions about impact on prevention**  No evidence of difference  **Do the reviewers agree with prevention conclusions?**  Yes |
| Costello (2008) | **Illness/events:**  No. of falls  Fall rates  No. of fallers | **Design and quality:** RCTs only – no QA  **Author conclusions about trustworthiness of evidence**  N/A – no assessment of quality  **How many studies are prevention SS based on?**  2 | Narrative synthesis | **Author conclusions about impact on prevention**  Evidence of positive impact  **Do the reviewers agree with prevention conclusions?**  Yes: No, quality of included studies is not known |
| Forbes (2008) | **Illness/events:**  Depression | **Design and quality:** RCTs only – with QA  **Author conclusions about trustworthiness of evidence**  High quality of evidence  **How many studies are prevention SS based on?** 1 | Meta-analysis | **Author conclusions about impact on prevention**  Evidence inconclusive  **Do the reviewers agree with prevention conclusions?**  Yes**:** insufficient evidence |
| Foster (2007) | **Illness/events:**  Depression  Anxiety  Psychological well-being  Health distress  Service use:  Physician/general practitioner visits  Days/nights spent in hospital | **Design and quality:** RCTs only – with QA  **Author conclusions about trustworthiness of evidence**  Concerns about quality of evidence  **How many studies are prevention Summary Statements based on?**  Illness/events: Depression, anxiety and psychological well-being, health distress = 9  Service use: Days/ nights spent in hospital = 6  Physician/ GP visits = 9 | Meta-analysis | **Author conclusions about impact on prevention**  Evidence of positive impact for illness/ events – depression and anxiety  No evidence of difference for illness/events – psychological well-being  No evidence of difference for number of visits to physician/ GP; number of days spent in hospital  Note: The interventions did not have any clinically important effect on depression or anxiety at six months follow-up  **Do the reviewers agree with prevention conclusions?**  Yes |
| Hand (2011) | **Illness/events:**  Psychological health | **Design and quality:** RCTs only – with QA  **Author conclusions about trustworthiness of evidence**  High quality of evidence  **How many studies are prevention SS based on?**2 | Narrative synthesis | **Author conclusions about impact on prevention**  Evidence of positive impact  **Do the reviewers agree with prevention conclusions?**  No: Reviewers are concerned about the quality of studies, the relevance of evidence for OT, and that no statistical information is provided to support the authors' claims about study findings |
| Harling (2008) | **Illness/events:**  Falls | **Design and quality:** RCTs only – with QA  **Author conclusions about trustworthiness of evidence**  High-quality evidence  **How many studies are prevention SS based on?**6 | Narrative synthesis | **Author conclusions about impact on prevention**  Evidence of positive impact  **Do the reviewers agree with prevention conclusions?**  Yes |
| Hauser (2010) | **Illness/events:**  Depressed mood | **Design and quality:** RCTs only – with QA  **Author conclusions about trustworthiness of evidence**  Concerns about quality of evidence  **How many studies are prevention SS based on?** 17 | Meta-analysis | **Author conclusions about impact on prevention**  Evidence of positive impact  **Do the reviewers agree with prevention conclusions?**  Yes |
| Hoey (2008) | **Illness/events:**  Psychological distress | **Design and quality:** RCTs – with QA  **Author conclusions about trustworthiness of evidence**  High-quality evidence  **How many studies are prevention SS based on?**  3 | Narrative synthesis | **Author conclusions about impact on prevention**  No evidence of difference  **Do the reviewers agree with prevention conclusions?**  Yes |
| Jain (2010) | **Illness/events:**  Depression  Anxiety  Negative mood | **Design and quality:** nRCTs and RCTs – with QA  **Author conclusions about trustworthiness of evidence**  High-quality evidence  **How many studies are prevention SS based on?** 8 | Narrative synthesis | **Author conclusions about impact on prevention**  Evidence inconclusive  **Do the reviewers agree with prevention conclusions?**  Yes |
| Lee (2007) | **Illness/events:**  Depression  Mood | **Design and quality:** RCTs – with QA  **Author conclusions about trustworthiness of evidence**  Concerns about quality of evidence  **How many studies are prevention SS based on?** 2 | Narrative synthesis | **Author conclusions about impact on prevention**  Evidence inconclusive  **Do the reviewers agree with prevention conclusions?**  Yes: Limited low- quality evidence |
| Legg (2007) | **Illness/events:**  Depression  Mood  **Service use:**  Use of institutional care | **Design and quality:** RCTs only – with QA  **Author conclusions about trustworthiness of evidence**  High-quality evidence  **How many studies are prevention SS based on?**  Service use: 3; Mood: 2 | Meta-analysis | **Author conclusions about impact on prevention**  Evidence of positive impact: the odds of a poor outcome were significantly lower in the participants who received occupational therapy  Evidence inconclusive: use of institutional care: data were incomplete and available for only a few studies and therefore the results from pooled analysis were inconclusive  Mood: There was a non-significant benefit in mood or distress scores for participants and carers  **Do the reviewers agree with prevention conclusions?**  Yes |
| Leung (2011) | **Illness/events:**  No. of falls | **Design and quality:** RCTs – with QA  **Author conclusions about trustworthiness of evidence**  High-quality of evidence  **How many studies are prevention SS based on?** 5 | Meta-analysis | **Author conclusions about impact on prevention**  Evidence of positive impact (health of older adults)  Evidence of harm (frail older adults)  Note: Positive impact was found when compared with no intervention; when compared with other exercise, the intervention findings were not significant at 26 and 52 weeks  **Do the reviewers agree with prevention conclusions?**  Yes |
| Lin (2011) | **Illness/events:**  Depression  Anxiety  Distress | **Design and quality:** RCTs only – with QA  **Author conclusions about trustworthiness of evidence**  Concerns about quality of evidence  **How many studies are prevention SS based on?** 8 | Meta-analysis | **Author conclusions about impact on prevention**  Evidence of positive impact  **Do the reviewers agree with prevention conclusions?**  Yes |
| Mayo-Wilson (2008) | **Illness/events:**  Physical health  Mortality  **Service use:**  Long-term institutional care | **Design and quality:** nRCTs and RCTs – with QA  **Author conclusions about trustworthiness of evidence**  Concerns about quality of evidence  **How many studies are prevention Summary Statements based on?** Long-term institutional care: 1**;** Physical health: 2 | Narrative synthesis | **Author conclusions about impact on prevention**  No evidence of difference: long-term service use  Evidence inconclusive: illness/events  **Do the reviewers agree with prevention conclusions?**  Yes: For illness/events outcomes  No: For use of long-term institutional care – authors make quite a strong claim, given that this is based on 1 study only |
| Michael (2010) | **Illness/events:**  Risk of falling  Number of falls  Increased falls | **Design and quality:** RCTs only – with QA  **Author conclusions about trustworthiness of evidence**  Concerns about quality of evidence  **How many studies are prevention Summary Statements based on?** 3 | Narrative synthesis | **Author conclusions about impact on prevention**  No evidence of difference: There was no evidence of increased falls or fallers, based on the 3 fair-quality trials that included home-hazard modification interventions  **Do the reviewers agree with prevention conclusions?**  No, the lack of clarity around the impact of this intervention renders the evidence inconclusive |
| Mont-gomery (2008) | **Illness/events:** Mental health  Depressive symptoms  Personal adjustment  Emotional health  **Service use:**  Use of institutional care | **Design and quality:** RCTs – with QA  **Author conclusions about trustworthiness of evidence**  High-quality evidence  **How many studies are prevention SS based on?**  Mental health: 2; Service use: 1 | Narrative synthesis | **Author conclusions about impact on prevention**  No evidence of difference: service use  Evidence inconclusive: depressive symptoms, personal adjustment, emotional health  **Do the reviewers agree with prevention conclusions?**  Yes: Evidence is inconclusive for mental health outcomes  No: Definitely concerned about conclusion on service use as strong claim based on 1 study only |
| O'Brien (2010) | **Illness/events:**  Psychological status | **Design and quality:** RCTs only – with QA  **Author conclusions about trustworthiness of evidence**  Concerns about quality of evidence  **How many studies are prevention SS based on?**2 | Meta-analysis | **Author conclusions about impact on prevention**  Evidence of positive impact  **Do the reviewers agree with prevention conclusions?**  Yes |
| Olazaran (2010) | **Illness/events:**  Mood | **Design and quality:** RCTs only – with QA  **Author conclusions about trustworthiness of evidence**  Concerns about quality of evidence  **How many studies are prevention SS based on?** 3 | Meta-analysis | **Author conclusions about impact on prevention**  1) Evidence of positive impact  2) Evidence inconclusive  **Do the reviewers agree with prevention conclusions?**  1) Yes: Sound meta-analytic evidence showing a positive impact of enriched group cognitive stimulation  2) Yes: Due to lack of studies the exercise and behaviour management intervention cannot currently be recommended |
| Padilla (2011) | **Illness/events:**  Depression  Anxiety | **Design and quality:** CTs and RCTs – with QA  **Author conclusions about trustworthiness of evidence**  Concerns about quality of evidence  **How many studies are prevention Summary Statements based on?**  1 | Narrative synthesis | **Author conclusions about impact on prevention**  No evidence of difference  **Do the reviewers agree with prevention conclusions?**  No: Although the single study providing evidence on this outcome was of high quality, reviewers feel that further evidence is needed to draw conclusions |
| Salter (2010) | **Illness/events:**  Depression  Distress  Mood status | **Design and quality:** RCTs only – with QA  **Author conclusions about trustworthiness of evidence**  Concerns about quality of evidence  **How many studies are prevention Summary Statements based on?** 8 | Narrative synthesis | **Author conclusions about impact on prevention**  Evidence inconclusive  **Do the reviewers agree with prevention conclusions?**  Yes: Agree that inconclusive – but think authors overstate the significance of the 1 trial with positive findings, particularly as this trial was one of the few which did not report concealment of allocation |
| Sawka (2010) | **Illness/events:**  Hip fractures | **Design and quality:** RCTs only – with QA  **Author conclusions about trustworthiness of evidence**  Concerns about quality of evidence  **How many studies are prevention SS based on?** 5 | Meta-analysis | **Author conclusions about impact on prevention**  Evidence of positive impact  **Do the reviewers agree with prevention conclusions?**  Yes |
| Tung-punkom (2012) | **Illness/events:**  Mental state | **Design and quality:** nRCTs and RCTs – with QA  **Author conclusions about trustworthiness of evidence**  Concerns about quality of evidence  **How many studies are prevention Summary Statements based on?** 1 | Narrative synthesis | **Author conclusions about impact on prevention**  Evidence inconclusive  **Do the reviewers agree with prevention conclusions?**  Yes |
| Yohannes (2010) | **Illness/events:**  Depression | **Design and quality:** nRCTs and RCTs – with QA  **Author conclusions about trustworthiness of evidence**  N/A – no assessment of quality  **How many studies are prevention Summary Statements based on?** 7 | Narrative synthesis | **Author conclusions about impact on prevention**  No evidence of difference: studies that investigated the benefits of exercise interventions on depression are inconclusive  **Do the reviewers agree with prevention conclusions?**  Yes |

##

## Satisfaction with services outcomes

| **Review** | **Satisfaction outcomes** | **Included studies** | **Review Methods** | **Conclusions** |
| --- | --- | --- | --- | --- |
| Allison (2011) | Satisfaction | **Design and quality**: RCTs only – with QA  **Author conclusions about trustworthiness of evidence**  Concerns about quality of evidence  **How many studies are satisfaction Summary Statements based on?** 4 | Narrative synthesis | **Author conclusions about impact on satisfaction**  Evidence of positive impact  **Do the reviewers agree with satisfaction conclusions?**  No: lack statistically significant differences |
| Legg (2007) | Satisfaction | **Design and quality**: RCTs only – with QA  **Author conclusions about trustworthiness of evidence**  Concerns about quality of evidence  **How many studies are satisfaction Summary Statements based on?** 2 | Meta-analysis | **Author conclusions about impact on satisfaction**  Evidence inconclusive:  **Do the reviewers agree with satisfaction conclusions?**  Yes |
| Mayo-Wilson (2008) | Satisfaction | **Design and quality**: CTs and RCTs – with QA  **Author conclusions about trustworthiness of evidence**  Concerns about quality of evidence  **How many studies are satisfaction Summary Statements based on?** 2 | Narrative synthesis | **Author conclusions about impact on satisfaction**  Evidence of positive impact; No evidence of difference  **Do the reviewers agree with satisfaction conclusions?**  Yes: Agree with authors’ cautious conclusions that intervention 'may' increase satisfaction |
| Montgomery (2008) | Satisfaction | **Design and quality**: CTs and RCTs – with QA  **Author conclusions about trustworthiness of evidence**  High-quality evidence  **How many studies are satisfaction Summary Statements based on?** 4 | Narrative synthesis | **Author conclusions about impact on satisfaction**  Evidence of positive impact  **Do the reviewers agree with satisfaction conclusions?**  Yes: Agree with cautiousness of authors’ conclusions about 'possible' increases in satisfaction |

## Safeguarding outcomes

| **Review** | **Safeguarding Outcomes** | **Included studies** | **Review Methods** | **Conclusions** |
| --- | --- | --- | --- | --- |
| Lindbloom (2007) | Nursing home staff attitudes towards elderly/ knowledge of elder abuse | **Design and quality**: CTs and RCTs – no QA  **Author conclusions about trustworthiness of evidence**  n/a- no assessment of quality  **How many studies are safeguarding Summary Statements based on?** 1 | Narrative synthesis | **Author conclusions about impact on safeguarding**  Evidence of positive impact  **Do the reviewers agree with safeguarding conclusions?**  No: On the strength of only one RCT, reviewers feel evidence is inconclusive |
